# Novel strategies for vancomycin-resistant *Enterococcus faecalis* biofilm control: bacteriophage (vB_EfaS_ZC1), propolis, and their combined effects in an ex vivo endodontic model

**DOI:** 10.1186/s12941-025-00790-y

**Published:** 2025-04-13

**Authors:** Toka A. Hakim, Bishoy Maher Zaki, Dalia A. Mohamed, Bob Blasdel, Mohamed A. Gad, Mohamed S. Fayez, Ayman El-Shibiny

**Affiliations:** 1 Center for Microbiology and Phage Therapy, Zewail City of Science and Technology, Giza, 12578 Egypt; 2 Microbiology and Immunology Department, Faculty of Pharmacy, October University for Modern Sciences and Arts (MSA), Giza, 11787 Egypt; 3 ESCMID Study Group on Biofilms (ESGB), Basel, Switzerland; 4 Department of Endodontics, Faculty of Dentistry, Suez Canal University, 4.5 Ring Road, Ismailia, 41522 Egypt; 5 Department of Endodontics, Faculty of Dentistry, Sinai University, Kantara-Shark, Ismailia, Egypt; 6 Vésale Bioscience, Vésale Pharmaceutica, 5310 Noville-Sur-Mehaigne, Belgium; 7 Faculty of Environmental Agricultural Sciences, Arish University, Arish, 45511 Egypt

**Keywords:** Antibiofilm, Endodontic treatment, Irrigation, Phage therapy, Propolis, *Saphexavirus*, Vancomycin-resistant *Enterococcus faecalis*

## Abstract

**Background:**

Endodontic treatment failures are predominantly attributed to *Enterococcus faecalis* (*E. faecalis*) infection, a Gram-positive coccus. *E. faecalis* forms biofilms, resist multiple antibiotics, and can withstand endodontic disinfection protocols*.* Vancomycin-resistant strains, in particular, are challenging to treat and are associated with serious medical complications.

**Methods:**

A novel phage, vB_EfaS_ZC1, was isolated and characterized. Its lytic activity against *E. faecalis* was assessed in vitro through time-killing and biofilm assays. The phage's stability under various conditions was determined. Genomic analysis was conducted to characterize the phage and its virulence. The phage, propolis, and their combination were evaluated as an intracanal irrigation solution against a 4-week *E. faecalis* mature biofilm, using an ex vivo infected human dentin model. The antibiofilm activity was analyzed using a colony-forming unit assay, field emission scanning electron microscopy, and confocal laser scanning microscopy.

**Results:**

The isolated phage, vB_EfaS_ZC1, a siphovirus with prolate capsid, exhibited strong lytic activity against Vancomycin-resistant strains. In vitro assays indicated its effectiveness in inhibiting planktonic growth and disrupting mature biofilms. The phage remained stable under wide range of temperatures (− 80 to 60 °C), tolerated pH levels from 4 to 11; however the phage viability significantly reduced after UV exposure. Genomic analysis strongly suggests the phage's virulence and suitability for therapeutic applications; neither lysogeny markers nor antibiotic resistance markers were identified. Phylogenetic analysis clustered vB_EfaS_ZC1 within the genus *Saphexavirus.* The phage, both alone and in combination with propolis, demonstrated potent antibiofilm effects compared to conventional root canal irrigation.

**Conclusion:**

Phage vB_EfaS_ZC1 demonstrates a promising therapy, either individually or in combination with propolis, for addressing challenging endodontic infections caused by *E. faecalis*.

**Supplementary Information:**

The online version contains supplementary material available at 10.1186/s12941-025-00790-y.

## Background

*Enterococcus faecalis (E. faecalis)* is a Gram-positive, opportunistic pathogen that causes several infections in the oral cavity, such as marginal periodontitis, dental caries, peri-implantitis, oral mucosal lesions, and root canal infections [1, 2]. The widespread occurrence of multidrug-resistant (MDR) *E. faecalis*, particularly vancomycin-resistant *E. faecalis* (VREF), has greatly hindered the effectiveness of traditional antibiotics in eliminating these infections. This represents an increased risk to global health [3, 4]. Consequently, combating the rise of MDR pathogenic bacteria is now a top priority for the World Health Organization (WHO).

*E. faecalis* infections can be severe due to its various virulence factors, including adherence, invasion, abscess formation, immune manipulation, and toxin secretion [5]. The genome of *E. faecalis* consists of antibiotic-resistance genes, which report over 25% of its total genetic content [6]. Furthermore, Antibiotic overuse and abuse accelerate the development of antibiotic-resistant bacteria [7]. Another cause contributing to antibiotic ineffectiveness is the development of bacterial biofilms [8]. Biofilms are highly resistant to antimicrobial treatments and immune responses, often exhibiting a hundred times greater persistence than planktonic cells [9, 10]. Bacterial biofilms play a crucial role in nearly all significant diseases in dentistry. Periodontitis presents a formidable challenge as it is a biofilm-mediated disease that withstands antibiotic treatments and host defenses [11, 12]. Besides, the primary biological cause of root canal diseases is the endodontic biofilm [13, 14], mainly comprised of *E. faecalis* commonly encountered in previously treated root canals [15].

Several studies have demonstrated a higher prevalence of *E. faecalis,* reaching up to 90% in unsuccessful endodontic therapy [2, 16]. Sodium hypochlorite (NaOCl) is commonly used during root canal treatment, but its effectiveness is limited by its potential to damage dentin and leave residual biofilms [17–23].

Hence, it is crucial to expedite the development of promising, non‐conventional, novel antimicrobial therapies [24], including natural products. Particularly phage-based products have the potential to reduce the impact of the burden of biofilm-induced MDR bacterial infections with minimal side effects [25]. Propolis, a resinous material gathered by bees, is valued for its potent antioxidant, anti-inflammatory, and antimicrobial qualities. These effects are attributed to bioactive compounds, including flavonoids, which inhibit bacterial RNA polymerase and interfere with the microbial cell wall or membrane, impairing cellular function and integrity.

Bacteriophages, also known as phages, are viruses that infect and replicate only within bacterial cells to combat bacterial pathogens [26, 27]. The ultimate success of phage therapy in clinical practice relies on compelling evidence of its safety and minimal side effect [28], is non-toxic for eukaryotic cells, and has excellent specificity for the bacterial host without affecting the normal microbiota [29]. Only virulent phages are suitable for phage therapy, replicating via the lytic cycle. Moreover, some phages can disrupt bacterial biofilms by producing tail-associated depolymerase activity [30]. The accessibility of isolating novel phages from diverse origins and the cost-effectiveness of producing phage preparations compared to manufacturing novel antimicrobial agents are other advantages of phage therapy [31].

Therefore, using phage therapy in Endodontics to improve root canal disinfection protocols was interesting. Limited studies were found in this area, though the direct effect of bacteriophage used as irrigation on *E. faecalis* biofilm was not previously studied [31–34]. Also, the previous studies separately evaluated the antibacterial effect of propolis [35] or bacteriophage [36] in endodontic infection. However, no study evaluated the antibacterial effect of propolis and bacteriophage when combined. We aimed to isolate and identify phages targeting and destroying MDR *E. faecalis*, including their biofilm. Subsequently, the isolated phage was characterized by sequencing and annotating the genome.

In this work, the main objective of the ex vivo experiment was to evaluate the antibacterial activity of the isolated phage and propolis extract as irrigants separately and in combination and compare this antibacterial efficiency with the traditionally used NaOCl irrigation. The antibacterial effect was tested against a mature biofilm of *E. faecalis* using an ex vivo human tooth-infected dentin model.

## Results

### Bacterial characterization and host range determination

**Fig. 1:**
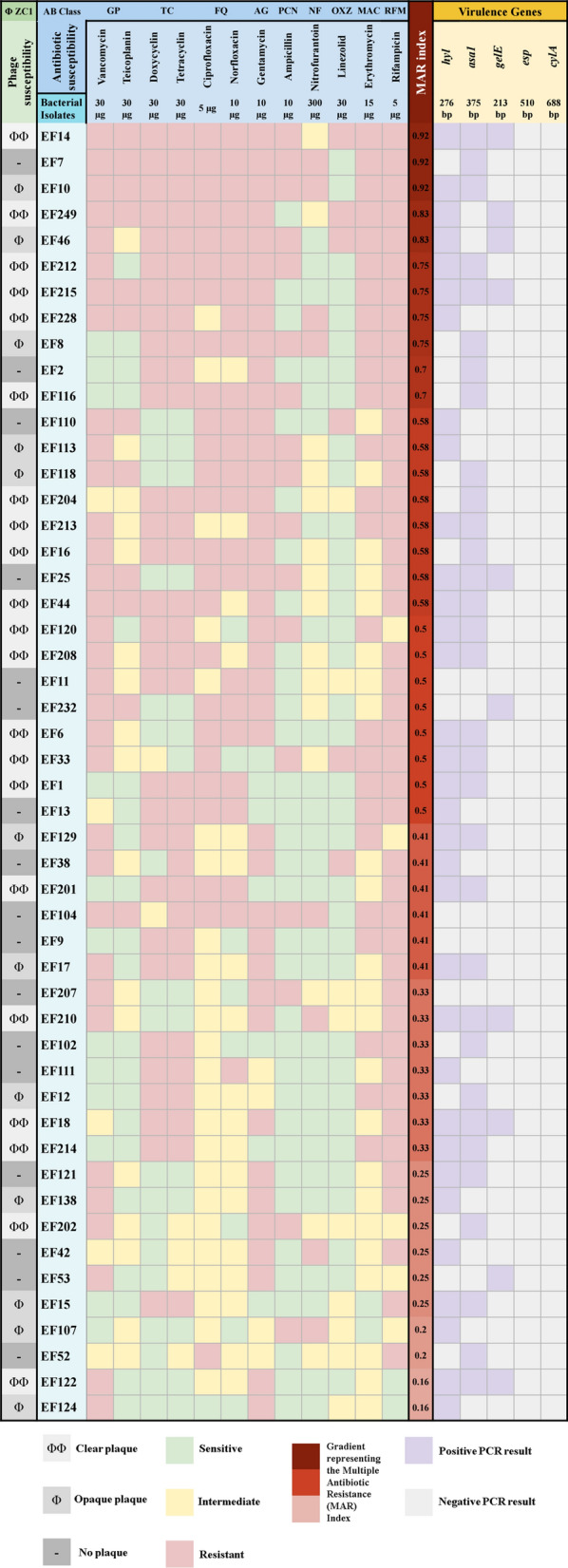
Heatmap illustrating the susceptibility to phage vB_EfaS_ZC1 and antimicrobial agents, and virulence gene profiles of 50 bacterial isolates. The left panel describes the host range of phage vB_EfaS_ZC1 visualized by plaque morphology: clear lysis (ΦΦ), opaque lysis (Φ), and no lysis (-) for each bacterial isolate. The middle panel represents the antimicrobial susceptibility profiles using color coding: green for sensitive, yellow for intermediate, and red for resistant isolates. A gradient heatmap in shades of red represents the Multiple Antibiotic Resistance (MAR) Index, with darker shades indicating higher indices. The right panel presents the PCR detection results for five virulence genes, with purple and light gray indicating positive and negative results, respectively

The bacterial isolates were identified as *E. faecalis*, forming a black complex on bile esculin. Subsequent Vitek analysis confirmed the identification with 99.9% accuracy as *E. faecalis*. PCR analysis further confirmed the identification of the 50 *E. faecalis* isolates, including the EF/14 strain, which exhibited a 941 bp band corresponding to the targeted amplified region of *ddl* gene of *E. faecalis.* Additionally, multiplex PCR was conducted targeting the five virulence genes **(**Fig. 1**)**. Among these, *hyl* and *asa1* were detected in most of the isolates, while *gelE* was detected in only a few isolates. Notably, *esp* and *cylA* were not detected in any of the isolates. A heatmap describes the susceptibility of all bacterial isolates to twelve antibiotics from nine classes. Most of the isolates demonstrated a high resistance profile to the tested antibiotics, with MAR indices of ≥ 0.2 and ≥ 0.5 observed in most of the isolates and ~ 50% of the isolates, respectively. The phage-isolating host (EF/14) had the highest MAR index (0.92), as it exhibited resistance to all tested antibiotics except Nitrofurantoin, to which it demonstrated intermediate susceptibility.

Phage vB_EfaS_ZC1 exhibited lytic activity against 33 bacterial isolates. These susceptible isolates displayed two distinct plaque morphologies: 21 isolates formed clear plaques, while 12 isolates formed opaque plaques. The remaining 17 isolates were not susceptible to phage vB_EfaS_ZC1 and exhibited no visible effect **(**Fig. 1**)**.

### Phenotypic characterization of phage vB_EfaS_ZC1

#### Phage stability

**Fig. 2:**
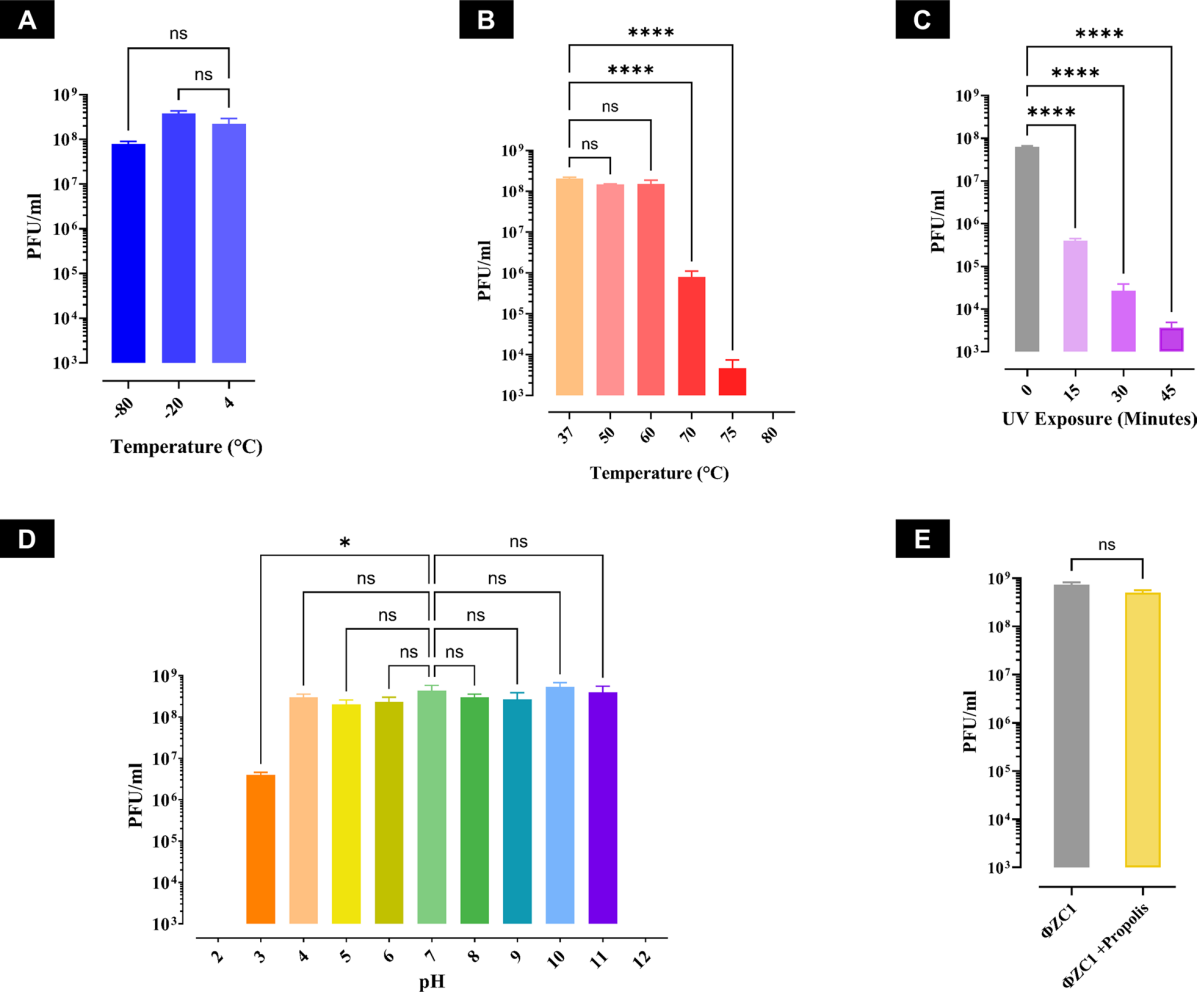
Stability assessment of phage vB_EfaS_ZC1 under various conditions. **A** Storage temperatures stability at − 80 °C, − 20 °C, and 4 °C. **B** Thermal stability of phage vB_EfaS_ZC1 at temperatures range from 37 to 80 °C. **C** Stability of the phage vB_EfaS_ZC1 under UV exposure assessed at an interval of 15 min for 45 min. **D** Stability of phage vB_EfaS_ZC1 across a pH range of 2–12. **E** Stability of phage vB_EfaS_ZC1 in the presence and absence of propolis (12 µg/mL), represented after a 3 h incubation period. Error bars represent the standard deviation, and statistical significance are indicated as ns for no significant difference (*P* ≥ 0.05), *indicates (*P* < 0.05), and ****indicates (*P* < 0.0001)

Phage vB_EfaS_ZC1 remained stable under storage conditions (− 80 °C, − 20 °C, and 4 °C), with no significant reduction in titer **(**Fig. 2A**).** Phage vB_EfaS_ZC1 also maintained stable at 37°C, 50°C, and 60 °C for an hour, with no significant difference in phage titer compared to 37 °C. However, the phage titer significantly decreased (*P* < 0.0001) at 70 °C, and likewise, it dropped below the detection limit at 80 °C **(**Fig. 2B**).** The titer of phage vB_EfaS_ZC1 significantly declined (*P* < 0.0001) after exposure to UV for 15 min, which continued to decline further by about tenfold every 15 min of exposure till the experiment end point at 45 min **(**Fig. 2C**).** Notably, phage vB_EfaS_ZC1 tolerated a broad range of acidic (pH 4–7) and alkaline (pH 7–11) conditions without any significant reduction in titer compared to neutral pH 7. However, a significant decrease in phage titer was observed at pH 3 (*P* < 0.05), and it became undetectable at pH 2 and pH 12 **(**Fig. 2D**)**. The propolis (12.5 µg/mL) did not affect the viability of phage vB_EfaS_ZC1 for 3 h at 4 °C **(**Fig. 2E**)**.

#### In vitro bacteriolytic activity and burst size

**Fig. 3:**
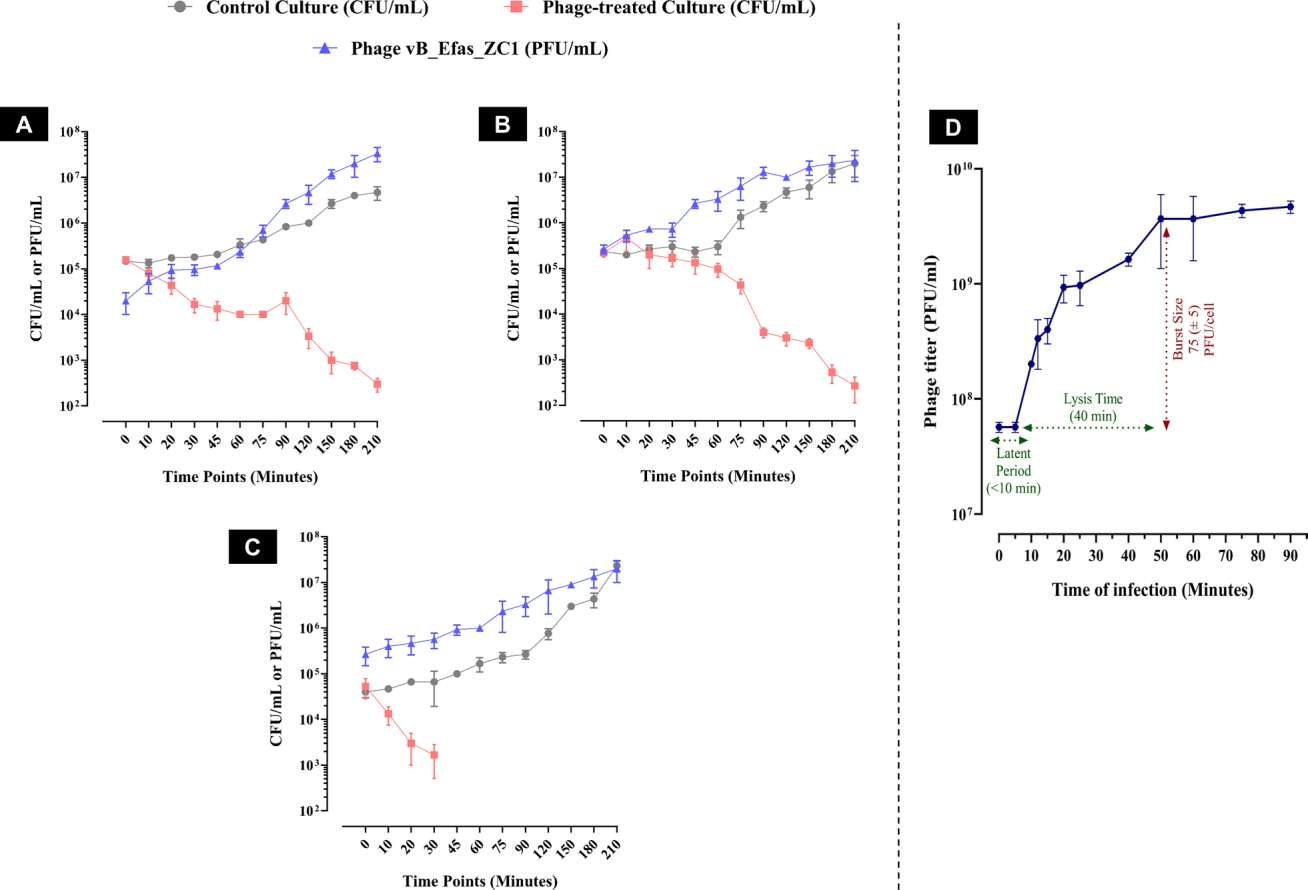
In vitro bacteriolytic dynamics of phage vB_EfaS_ZC1 at different MOIs. The three panels (**A**, **B**, and **C**) represent EF/14 cultures infected with phage vB_EfaS_ZC1 at MOIs 0.1, 1, and 10, respectively. These panels illustrate bacterial counts (CFU/mL) and phage titer (PFU/mL) over a period of 210 min. **D** One-step growth curve of phage vB_EfaS_ZC1 at MOI 0.1

Phage vB_EfaS_ZC1 demonstrated potent bacteriolytic activity against the EF/14 bacterial host at different MOIs (0.1, 1, and 10), assessed over 210 min **(**Fig. 3A–C**)**. The results revealed a reduction in EF/14 bacterial growth lysed by phage vB_EfaS_ZC1 compared to the untreated bacterial culture at the three tested MOIs At MOIs 0.1 and 1, the surviving bacteria in the phage treated culture continued to decline by approximately 3 log till the end of the experiment at 210 min **(**Fig. 3A and 3B, respectively**)**, while at an MOI of 10, the bacterial titer dramatically reduced within 30 min and remained undetectable till the end of the experiment **(**Fig. 3C**)**. Remarkably, the challenged bacteria did not resist the phage at all tested MOIs.

Phage vB_EfaS_ZC1 replication was investigated using a one-step growth curve at MOI of 0.1. The analysis revealed an estimated latent period of < 10 min, followed by a lysis time of 40 min. This resulted in a burst size of approximately 75 (± 5) PFU per infected host cell **(**Fig. 3D**)**.

#### Antibiofilm activity of phage vB_EfaS_ZC1

**Fig. 4:**
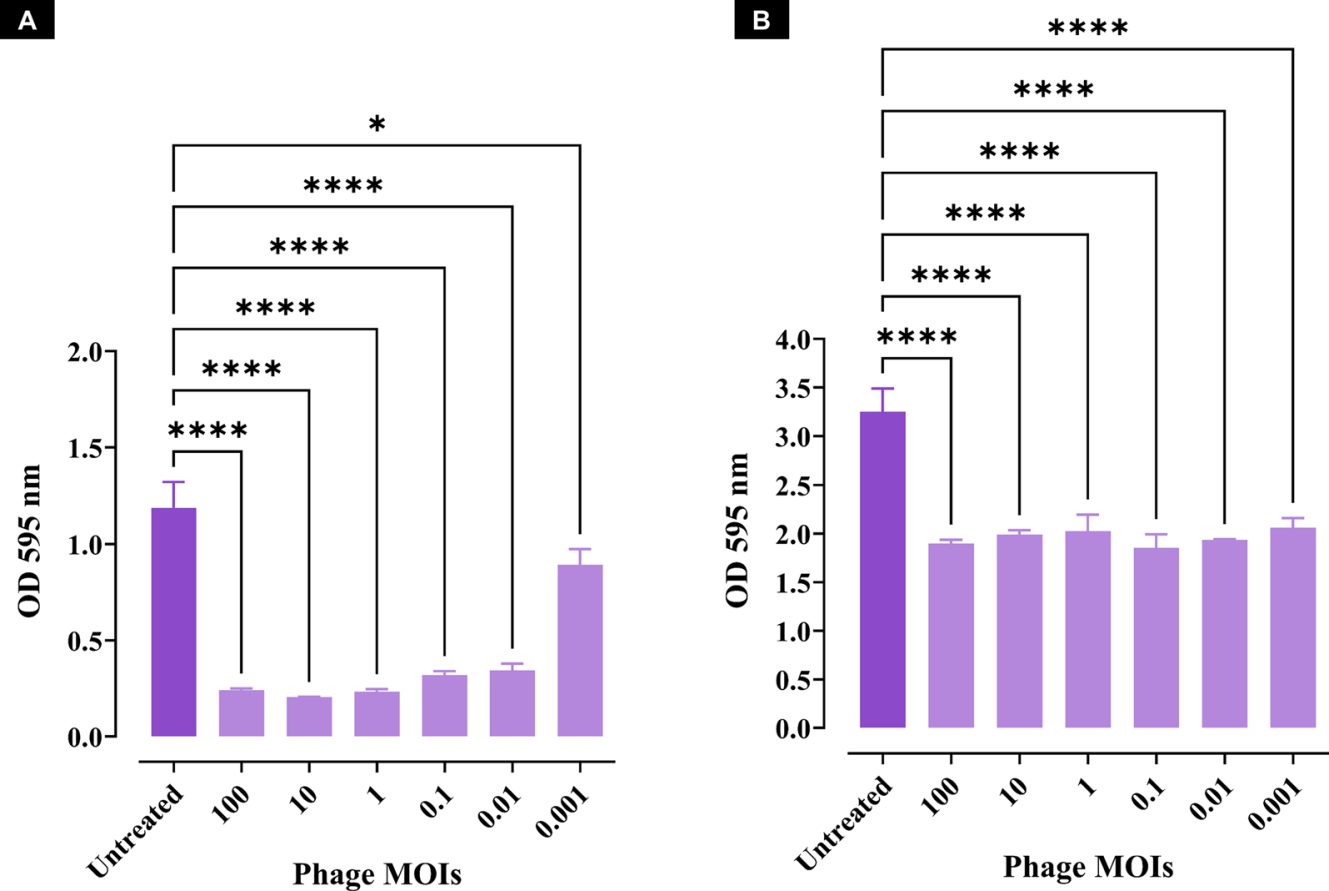
Antibiofilm activity of phage vB_EfaS_ZC1 at different MOIs. **A** Quantification of biofilm inhibition in bacterial cultures treated with phage vB_EfaS_ZC1 at MOIs (0.001–100), measured as OD 595 nm. **B** Quantification of biofilm clearance in preformed biofilms treated with phage vB_EfaS_ZC1 at MOIs (0.001–100), measured as OD 595 nm. Statistical significance between phage-treated groups and the untreated group is indicated * and ****which represent *P* < 0.05 and *P* < 0.0001, respectively

Phage vB_EfaS_ZC1 significantly inhibited bacterial biofilm development. MOIs ranging from 0.01 to 100 demonstrated comparable results with high significance (*P* < 0.0001), while an MOI of 0.001 also significantly inhibited the biofilm formation (*P* < 0.05) compared to the untreated bacterial culture **(**Fig. 4A**)**. The minimum infectious dose of MOI 0.01 was identified as the lowest MOI that exhibited the most potent inhibitory impact on biofilm production. Furthermore, in the biofilm clearance assay, all tested MOIs (100–0.01 demonstrated comparable efficacy in significantly clearing mature biofilms (*P* < 0.0001) compared to the untreated group **(**Fig. 4B**)**. The antibiofilm results underscore the potent activity of phage vB_EfaS_ZC1 against bacterial biofilm.

#### Morphology of phage vB_EfaS_ZC1

**Fig. 5:**
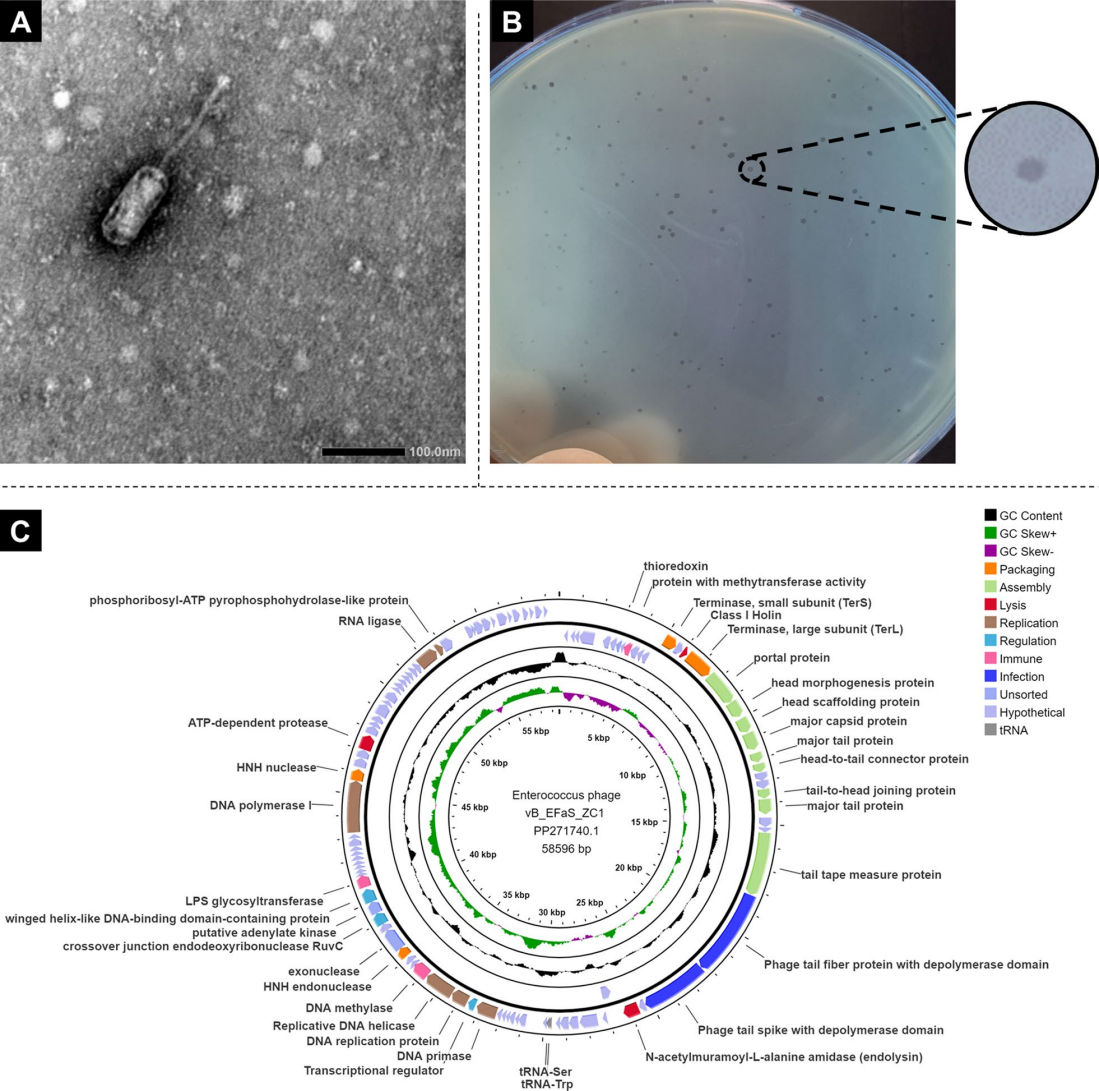
Morphological characteristics of phage vB_EFaS_ZC1. **A** Transmission electron micrograph of phage vB_EFaS_ZC1 captured using TEM **B** Phage vB_EFaS_ZC1 plaques formed in double-layer agar plates on the EF/14 lawn.** C** Circle map visualization of phage vB_EfaS_ZC1 genome. Colored segments represent coding sequences categorized by predicted function: orange (genome packaging), light green (assembly of virion proteins), red (lysis), brown (replication), turquoise (regulation), pink (immune), blue (infection), light blue (unsorted/hypothetical), and grey (tRNA gene). The middle circle displays GC content (black). The inner circle shows a GC skew with green for the positive skew and magenta for the negative skew

Phage vB_EfaS_ZC1 exhibited a prolate capsid with approximate dimensions (length ~ 102 nm and width ~ 42 nm) and a long, non-contractile tail measuring around ~ 133 nm, as observed by TEM **(**Fig. 5A**)**. The identified structural features are characteristic of the Siphovirus morphotype, as defined by the International Committee on Taxonomy of Viruses (ICTV) classification in its ninth report [37]. Phage vB_EfaS_ZC1 formed small, well-defined, and circular plaques on the bacterial lawn of EF/14, indicating efficient bacterial lysis and a lytic phage **(**Fig. 5B**)**.

### Genome and phylogeny analysis

The obtained reads were of length between 35 and 326 bases. The reads were successfully assembled into one contig with an average read depth and coverage of 215x. The size of the assembled contig is 58,596 bp, with an average GC content of 39.8%. The phage genome annotation revealed 102 ORFs and 2 tRNA genes (Fig. 5C and Supplementary Table S2). The tRNAs carry anticodons for tryptophan (Trp) and serine (Ser). The predicted ORFs included 11 genes that encode assembly (virion structural) proteins, 4 genome packaging genes, 3 lysis genes, and 69 hypothetical proteins. Depolymerase domains were predicted with > 90% confidence in ORFs 30 and 31, annotated as tail fiber and tail spike proteins, respectively. BLASTp analysis revealed that the N-terminal domain of the tail spike protein (ORF 31), putatively a hyaluronidase, shares high similarity (100% coverage, > 90% identity, and e-value = 0) with most saphexaviruses (Supplementary Fig. S3, Table S3).

ORF 15 is predicted to encode a class I holin based on high similarity in the amino acid sequence (100% coverage, > 98% identity, and e-value < 10^–44^) to known holins from closely related phages in the GenBank database and the predicted protein topology by DeepTMHMM. The predicted topology indicates class I features, including three transmembrane domains flanked by a periplasmic N-terminus and a cytoplasmic C-terminus (Supplementary Figure S4). The genomic analysis of phage suitability for therapy did not find any markers for AMR, virulence, lysogeny, and DGR systems.

**Fig. 6:**
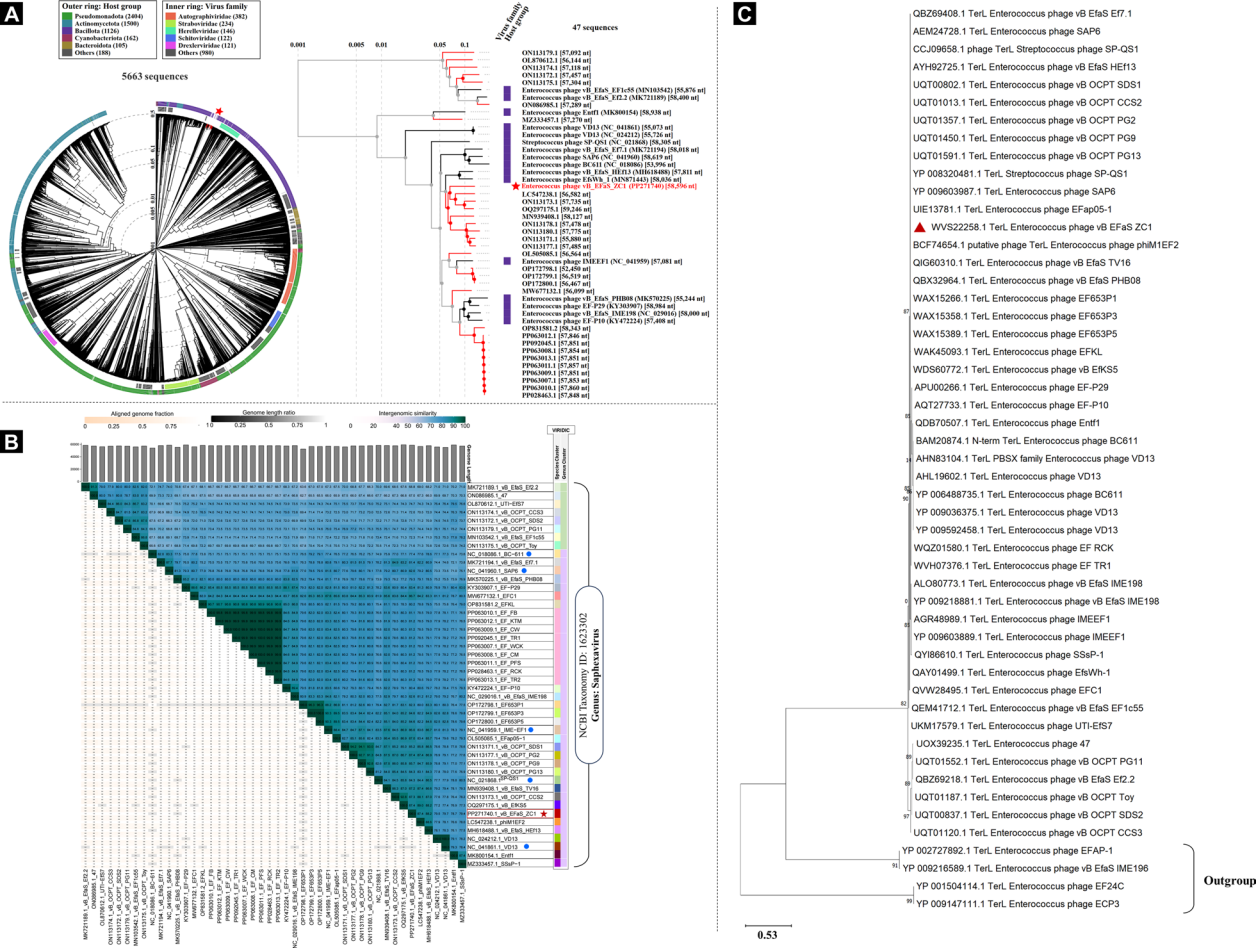
Phylogenetic analysis of phage vB_EfaS_ZC1. Three panels represent the evolutionary relationships of phage vB_EfaS_ZC1: **A** A proteomic tree with the phage marked by a red star, the circular tree comparing vB_EFaS_ZC1 to all phages in the ViPtree database, and the rectangular tree highlighting closely related phages with high ViPtree similarity scores (SG > 0.6). **B** VIRIDIC heatmap visualizing the intergenomic similarity of vB_EFaS_ZC1 (red star) to closely related phages, all members of the *Saphexavirus* genus based on NCBI taxonomy; *Saphexaviruses* in ICTV marked by blue circles. The heatmap represents clustering at genus and species levels. **C** A maximum likelihood phylogenetic tree comparing the terminase large subunit (TerL, core protein) of vB_EfaS_ZC1 (red triangle) with homologs from other closely related Saphexavirus phages. Four phages with low SG scores (< 0.6) to vB_EfaS_ZC1 were chosen as an outgroup. Phylogeny.fr was used for tree construction

Proteomic analysis using ViPtree (Fig. 6A, Supplementary Table S4) classified vB_EfaS_ZC1 with phages that infect bacteria of phylum Bacillota, but these phages have not been assigned to a specific family yet. Additionally, VIRIDIC analysis clustered phage vB_EfaS_ZC1 based on overall genome similarity as a unique species within the genus *Saphexavirus* (Fig. 6B). Likewise, the maximum likelihood tree of the core protein terminase (large subunit) grouped phage vB_EfaS_ZC1 with members of the genus *Saphexavirus* **(**Fig. 6C**).** These analyses strongly suggest that vB_EfaS_ZC1 is a novel phage which is related to *Saphexaviruses* genus.

### MIC and MBC of propolis

Propolis demonstrated potent antibacterial activity against EF/14 bacteria with a 12.5 µg/mL MIC. While a higher concentration of 25 µg/mL for MBC exhibited a bactericidal effect, confirming its efficacy in eliminating viable bacteria.

### Time Killing Curve of Phage vB_EfaS_ZC1 and Propolis

**Fig. 7:**
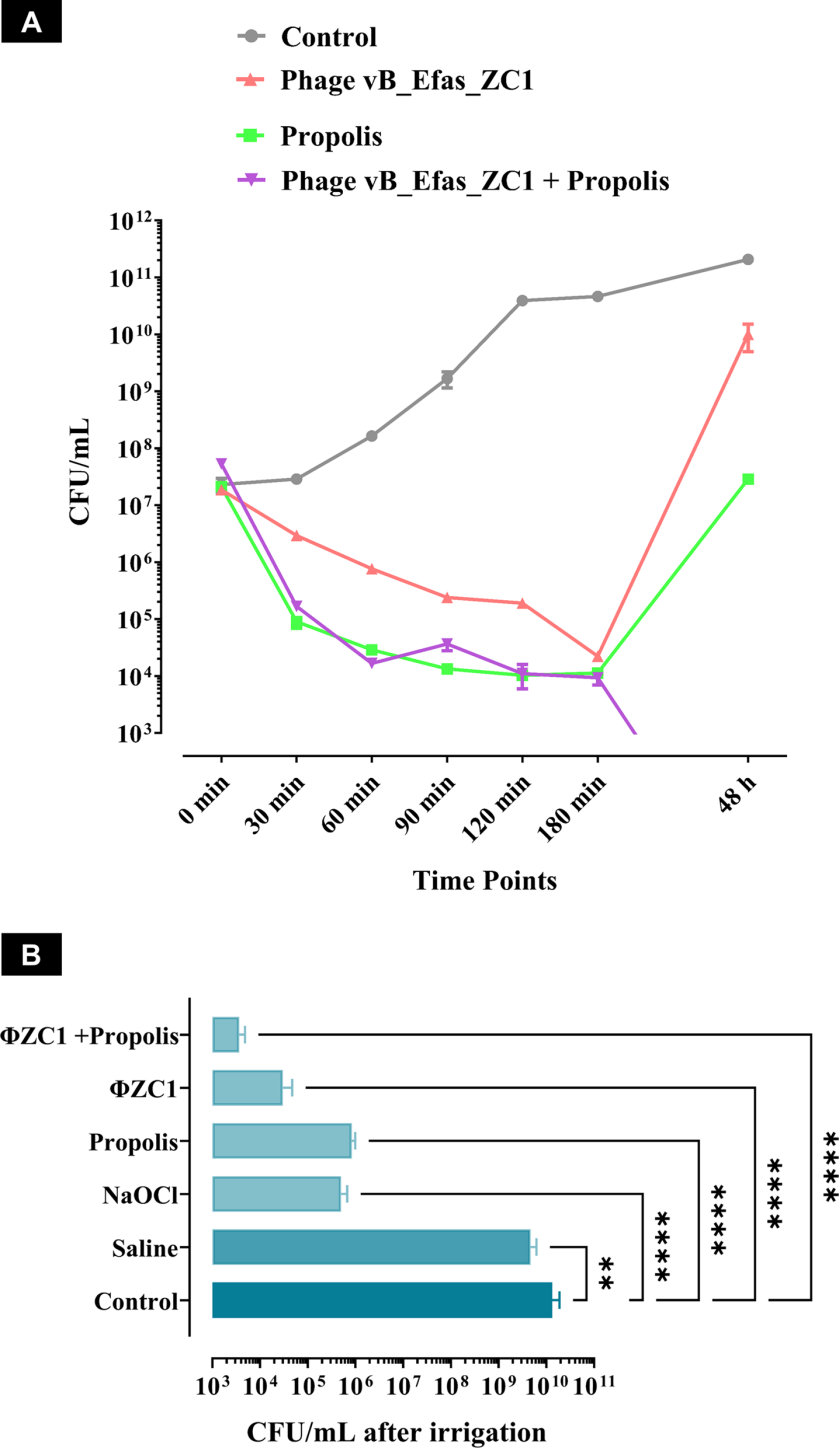
Evaluation of the antibacterial efficacy of phage, propolis, and their combination against EF/14 bacteria in planktonic and biofilm forms. **A** Time-killing curve of EF/14 culture treated with phage vB_EfaS_ZC1, propolis, and their combination over 48 h. **B** Efficacy of different irrigation treatments against EF/14 biofilms on dentin slices. The statistical significance of the reduction in bacterial count (CFU/mL) after different irrigation treatments compared to the control (untreated) group is represented by ** for *P* < 0.01 and **** for *P* < 0.0001

The time-killing curve analysis demonstrated that all tested treatments (phage, propolis, and combination of both) dramatically reduced bacterial culture. Meanwhile, the treatments included the phage or propolis alone exhibited bacterial regrowth after 180 min. Notably, the combination treatment (phage vB_EfaS_ZC1 and propolis) reduced bacterial growth below the detectable limit after 180 min and until the experiment end point (48 h). The combination demonstrated a more potent and sustained effect against *E. faecalis* **(**Fig. 7A**)**.

### Viable Bacterial Count Assessment After Irrigation of Dentin Slices

All treated groups exhibited extremely statistically significant differences (*p* < 0.0001) in CFU count, while the saline group demonstrated a very significant difference (*p* < 0.01) compared to the control group. The group treated with a combination phage vB_EfaS_ZC1 and propolis irrigation resulted in the lowest bacterial counts **(**Fig. 7B**)**. Total bacterial counts are presented as means and standard deviations (SDs) for the number of EF/14 colonies remaining after each irrigation.

### FE-SEM and CLSM analysis for tested irrigation

**Fig. 8:**
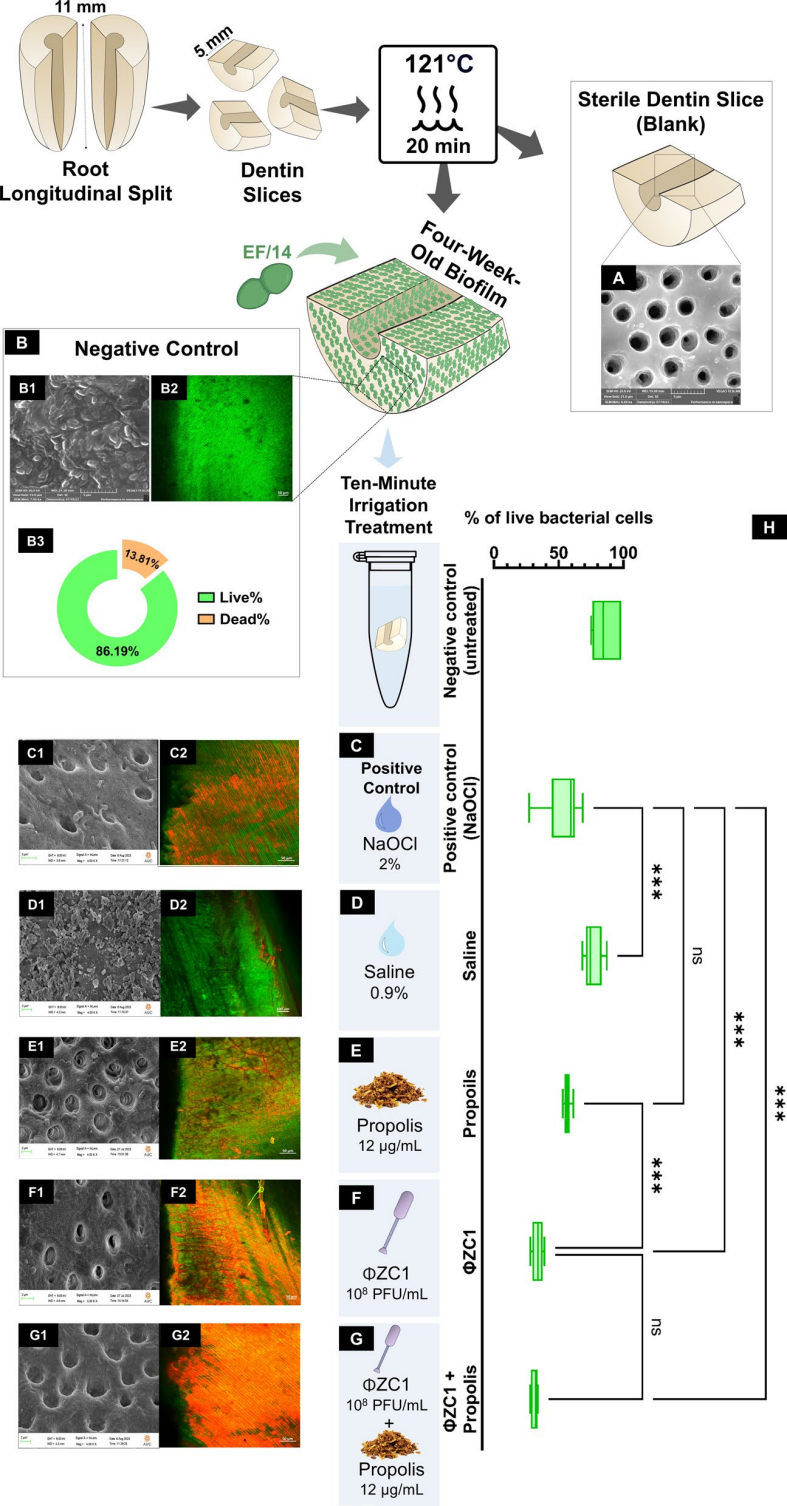
Characterization of EF/14 biofilm morphology and viability following irrigation treatments in ex vivo model of root dentin slices. **A** Sterilized blank group without biofilm, while (**B**) contained a four-week-old biofilm without any treatment. (**C- G**) underwent different irrigated treatments: (**C**) was treated with 2% Sodium Hypochlorite, (**D**) with 0.9% saline, (**E**) with propolis, (**F**) with phage vB_EfaS_ZC1, and (**G**) with a combination of phage vB_EfaS_ZC1 and propolis. These groups are represented in the corresponding figure. **B1-G1** Qualitative analysis was observed by FESEM images of the EF/14 biofilm after 10 min of different irrigation treatments. **B2–G2** Quantitative analysis by CLSM illustrating EF/14 biofilms (green: live bacteria; red: dead bacteria) on dentin slices after 10 min of different irrigation treatments. **H** Box plots demonstrate the percentage of live bacterial cells under different irrigation treatments. The median and interquartile range (n = 6) are represented for each group, Statistical significance is indicated by *** for *P* < 0.001, and ns indicates no significant difference.

The sterilized dentin slice, as verified by FE-SEM, confirmed the removal of the smear layer, revealing clear intratubular dentin free from any bacterial contamination (Fig. 8A**)**. Following a four-week incubation period with EF/14, the FE-SEM image demonstrated the successful formation of a biofilm, effectively blocking the dentinal tubules (DT) and mimicking the endodontic environment (Fig. 8B1). Additionally, CLSM analysis corroborated a high percentage of viable bacterial cells, further validating the establishment of a mature *E. faecalis* biofilm (Fig. 8B2-B3). FE-SEM analysis of the 2% NaOCl group revealed residual biofilm with some remnants of adherent biofilm on the dentin surface (Fig. 8C1). CLSM analysis demonstrated dead bacterial cells (red) scattered amongst the residual biofilm structure (Fig. 8C2). The saline group revealed extensive biofilm coverage on the root canal dentin surface by FE-SEM analysis (Fig. 8D1). Conversely, CLSM analysis exposed intact biofilm with a high abundance of viable bacteria (Fig. 8D2). The propolis treatment, while effective in killing bacteria, left behind residual biofilm on the dentin surface. This was confirmed by both FE-SEM and CLSM analysis (Fig. 8E1–E2). FE-SEM analysis of phage vB_EfaS_ZC1 indicates biofilm disruption on the dentin surface (Fig. 8F1). CLSM analysis for phage vB_EfaS_ZC1 demonstrates extensive biofilm eradication with dead bacterial cells (Fig. 8F2). The dentin surface of the combination group exhibited highly disruption biofilm, as a clear smear layer, with open dentinal tubules and absence of bacteria (Fig. 8G1), the combined phage vB_EfaS_ZC1 and propolis treatment revealed the most effective disinfection outcome. CLSM analysis demonstrates total eradication of live bacteria (Fig. 8G2). The dark background distinguishes non-fluorescent material.

Saline, phage vB_EfaS_ZC1, and the combination of phage vB_EfaS_ZC1 and propolis significantly reduced (*P* < 0.001) bacterial cells when compared to the positive control group (NaOCl). Importantly, no significant difference was observed between the propolis and NaOCl groups, also no significant difference between phage vB_EfaS_ZC1 and its combination with propolis (Fig. 8H). Among all tested irrigation treatments, the combination of phage vB_EfaS_ZC1 and propolis demonstrated the greatest reduction in live bacterial cells, which indicates the effectiveness of this combination as an endodontic disinfectant.

## Discussion

*E. faecalis* is the most prevalent pathogen in root canal infections, particularly, vancomycin-resistant and biofilm-forming strains that pose significant challenges due to treatment failure and recurrent infections [38–40]. While chlorhexidine and sodium hypochlorite are commonly used as endodontic irrigants, they have limitations, including a lack of residual efficacy and potential cytotoxicity to periapical tissues [2, 41]. To address these limitations, we investigated the application of bacteriophages against VRE.

Phage vB_EfaS_ZC1 successfully reduced the growth of MDR resistant *E. faecalis* isolate at MOI 0.1, 1, and 10. The short latent period and medium burst size observed for phage vB_EfaS_ZC1 fall within the range reported for many other *E. faecalis* phages [94, 96, 99, 102]. Moreover, phage vB_EfaS_ZC1 effectively reduced *E. faecalis* biofilms in an ex vivo model mimicking the complexities of a root canal infection. Additionally, the combination of vB_EfaS_ZC1 with propolis demonstrated enhanced antibacterial activity against planktonic bacteria compared to phage monotherapy, and this combination inhibited the emergence of phage-resistant mutants for up to 48 h. However, the combination and phage monotherapy had a comparable effect in the ex vivo model, potentially due to propolis's limited penetration into mature biofilms.

Phage vB_EfaS_ZC1 was evaluated based on the key characteristics of an ideal therapeutic phage, which mainly include strict lytic life mode, safety profile, and broad host range [42, 43]. No lysogeny markers, virulence, and antibiotic resistance genes were identified in the phage vB_EFaS_ZC genome, which strongly suggests the phage's suitability for therapeutic applications. Moreover, the phage demonstrated high efficacy in eliminating the target pathogen, even the most challenging multidrug-resistant and biofilm-forming strain. Additionally, the phage has a relatively broad host range of clinical *E. faecalis* isolates.

Phylogenetic analyses assign phage vB_EfaS_ZC1 to the genus *Saphexavirus.* In this section, the phage stability, antibiofilm activity, and replication dynamics are compared against representative *E. faecalis* phages from *Saphexavirus* genus IME-EF1[44], vB_EfaS_HEf13 [45], vB_EfaS_PHB08 [46], and EF-P29 [47], other siphoviruses SFQ1 [42], Ef212 [48], myoviruses vB_Efa29212_3e [36], and podoviruses vB_ZEFP [32]. Phage vB_EfaS_ZC1 tolerated a broad temperature range (−80°C to 60°C) over an hour which is comparable to the demonstrated stability of phages vB_EfaS_PHB08 [46], vB_EfaS-271 [49], EPC, and EPE [50]. Meanwhile, phages vB_EfaS_HEf13 [45], SFQ1 [42], and vB_EfaS-SRH2 [51] were stable at lower temperatures. This remarkable temperature tolerance suggests that phage vB_EfaS_ZC1 withstands harsh environmental conditions during storage and transportation.

Additionally, phage vB_EfaS_ZC1 maintained its viability under acidic and alkaline conditions (pH 3–11) with a limited reduction in viability after 24 h of exposure to pH 3. Consequently, phage vB_EfaS_ZC1 had comparable pH stability to phage vB_EfaS_HEf13 [45]. While other phages, vB_Efa29212_2e and vB_Efa29212_3e [36] demonstrates stability at a narrower pH range. Given the characteristically acidic environment of endodontic infections [52], the stability of phage vB_EfaS_ZC1 at low pH strongly supports its potential efficacy in treating this condition.

Phage vB_EfaS_ZC1 exhibited potent antibiofilm activity in both in vitro and ex vivo models, consistent with the antibiofilm properties of other *Enterococcus* phages, including saphexaviruses vB_EfaS_PHB08 [46] and vB_EfKS5 [53] and other siphoviruses vB_EfaS-271 [99], vB_EfaS-SRH2 [51], and SA14 [102]. Depolymerase domains were identified within the tail fiber and tail spike proteins of phage vB_EfaS_ZC1. The latter suggests that the depolymerase could facilitate phage infection by degrading the biofilm matrix and penetrating the *Enterococcus* capsule to reach bacterial cell receptors [54].

Endodontic and periodontal biofilms, composed of multiple bacterial species, are challenging to treat. Several strategies have been proposed to prepare multi-species bacteriophage and mitigate bacterial resistance [55]. Phage cocktails targeting diverse bacteria can enhance antimicrobial efficacy against such biofilms, significantly lowering the chances of bacteria developing resistance [56]. Moreover, genetically modified phages offer another solution by targeting multiple bacterial receptors and disrupting shared biofilm components [57]. Complementary treatments like propolis, a natural antibacterial agent, act on bacterial membranes or cell walls and cause functional and structural damage [58]. Combining propolis with phage therapy may broaden the treatment spectrum, reducing the need for species-specific phage cocktails and providing a versatile approach to managing complex biofilms.

One of the limitations of this study is the absence of a preclinical animal model to evaluate potential side effects or immune responses associated with using phages as endodontic disinfectants. Although we conducted ex vivo experiments using extracted human teeth, these models do not fully replicate the complex host environment, including potential immune recognition of phages as non-self antigens.

Localized administration of phages, particularly through oral delivery, has the potential to confine treatment to the infection site and reduce the likelihood of significant immunogenicity [59]. In contrast, systemic administration can activate both innate and adaptive immune systems, often resulting in the production of anti-phage antibodies [60]. For instance, Sarker et al*.* [61], demonstrated the safety and the efficacy of oral phage therapy in children with acute bacterial diarrhea, reported no adverse events. However, other studies revealed that the phage interaction with the mammalian immune system can elicit humoral immune responses to produce antiphage antibodies with phage-neutralizing activity [62–64].

Further research using preclinical animal models is essential to investigate potential immune responses, including the generation of anti-phage antibodies and other localized or systemic effects, to ensure the safety and efficacy of phage-based therapies in endodontic applications. Additionally, exploring the synergistic effects of phage cocktails or combinations with other antimicrobial agents offers promising avenues for combating MDR *E. faecalis* in complex oral environments.

## Conclusions

This study identifies and describes a promising phage for therapy to address the persistent challenge of endodontic infections caused by vancomycin-resistant *Enterococcus faecalis*. Characterization of the novel phage vB_EfaS_ZC1 revealed its broad-spectrum efficacy and ability to eradicate mature biofilms. The phage’s robust stability under various environmental conditions enhances its potential for practical application. Moreover, the effects observed with propolis demonstrate the potential for developing innovative and effective endodontic treatment strategies. As antibiotic resistance and the ability of bacteria to form biofilms become increasingly prevalent, phage-based therapies like vB_EfaS_ZC1 emerge as compelling alternatives to traditional treatments in endodontic care. Consequently, phage therapy holds great promise as a transformative approach to endodontic treatment, with potential implications for addressing the broader challenge of antimicrobial resistance.

## Material and methods

### Bacterial isolates and preservation conditions

Fifty clinical bacterial isolates were gifted from the endodontic clinic at the Faculty of Dentistry, Suez Canal University, Egypt, to the Center of Microbiology and Phage Therapy at Zewail City for Technology and Science. The bacterial isolates were cultured on differential selective media Bile Esculin Azide agar (MAC; Oxoid, UK). The VITEK MS system (bioMérieux, Marcy l'Étoile, France) was used to identify the isolates of some of the acquired *E. faecalis* isolates, followed by identification through API 20 STREP strip systems (bioMérieux, Cairo, Egypt) [50]. One colony was inoculated into 1 mL of TSB (Oxoid, UK) with 20% glycerol and kept at −80°C for preservation.

### Bacterial confirmation and identification of virulence genes by polymerase chain reaction (PCR)

The bacterial identification was assessed by amplifying a conserved region of a specific gene *ddl *_*E. faecalis*_. PCR reaction was conducted using a mixture of 25 μL of Master Mix, one microliter of each forward and reverse primer, 5 μL of bacterial DNA obtained through the (QIAamp DNA Mini Kit). Finally, the remaining volume up to 25 μL was completed by nuclease-free water [65, 66]. Furthermore, multiplex colony PCR was performed to detect the presence of five virulence genes (*asa1*, *cylA*, *esp*, *gelE*, and *hyl*) in *Enterococci*, known to be key determinants of bacterial pathogenesis [67, 68]. Specific primer sets were used to target these genes (Supplementary Table S1), with product sizes varying to allow for clear differentiation by agarose gel electrophoresis.

### Antibiotic Susceptibility Profile

The antimicrobial susceptibility assay was conducted on 50 *E. faecalis* isolates using disk diffusion methods according to the Clinical and Laboratory Standards Institute 2022 recommendations (CLSI) [69]. Briefly, the diameter of the clear zone around the discs was interpreted as either resistant, intermediate, or susceptible according to the breakpoints of CLSI 2022. The suitability of each *E. faecalis* strain was tested on 12 discs of antibiotics (belonging to 9 different classes), such as Vancomycin (VA; 30 μg), Linezolid (LZD; 30 μg), Erythromycin (E; 15 μg), Teicoplanin (TEC; 30 μg), Rifampicin (RD; 5 μg), Nitrofurantoin(F; 300 μg), Ampicillin (AM; 10 μg), Doxycycline (DO; 30 μg), Tetracycline (TE; 30 μg), Gentamycin (CN;10 μg), Ciprofloxacin (CIP; 5 μg), and Norfloxacin (Nor; 10 μg) [32, 70]*.* Based on the resistance profile of each isolate, the Multiple Antibiotic Resistance (MAR) index was calculated, as described previously [71].

### Isolation, purification, and propagation of phage

Phages targeting *E. faecalis* were isolated from different sewage samples that were collected from different sites in Giza, Egypt. The sewage sample was mixed with *E. faecalis* cultures and fresh TSB in a centrifuge tube for enrichment. Afterward, the mixture underwent incubation in a shaker incubator at 100 rpm for 3 h at 37 °C [72]. Then, the enriched sample was centrifuged at 6163×g for 15 min at 4 °C. The supernatant (phage lysate) was filtered using a 0.45 µm porous Polyethersulfone syringe filter (Standard Membrane Filtration Limited, China) [73]. For phage screening, the double layer agar (DLA) method and spotting test were used as previously described [74], then plates were incubated at 37 °C for 24 h. A clear phage plaque was chosen for further characterization using *E. faecalis* (EF/14) as a bacterial host. The purification of phage plaque was accomplished by repeating the process of isolating a single plaque seven times. Briefly, each single plaque was collected and suspended in 200 µL sodium magnesium (SM) buffer and kept for 2 h to elute the entrapped phages. Next, the phage was amplified by using the plate lysate method [75], and the amplification titer was determined as a plaque-forming unit (PFU) using a spotting assay [76] of serially diluted phage lysate. The amplified phage was filtered using a syringe filter with 0.22 µm porous cellulose acetate and stored in SM buffer at 4 °C.

### Host Range and Lytic Profile

The host range and lysis efficiency of the purified phage were evaluated against *E. faecalis*. A total of 10 μL of purified phage was spotted in triplicates on 50 different bacterial lawns of *E. faecalis* on TSA plates with a double agar layer (0.5% Bacto Agar) containing exponentially growing bacterial culture. The plates were kept at 37°C overnight and inspected for plaque formation [77].

### Phage stability

The phage was serially diluted and spotted on a bacterial culture EF/14 to evaluate its temperature, UV, and pH stability. The temperature stability of the phage was evaluated over an hour of incubation at various temperatures (− 80, − 20, 4, 37, 50, 60, 70, 75, and 80 °C). The initial titer for phage aliquots was set to 10^8^ PFU/mL at each temperature. The phage was exposed to UV exposure for 15, 30, and 45 min to assess UV stability. Furthermore, the SM buffer at different pH levels ranging from 2 to 12 using 0.1 M NaCl and 0.1 M HCl to test the stability of the phage for 24 h. The phage titer for each pH was measured after the incubation period.

Moreover, the phage viability in the presence of propolis was evaluated [78]. In brief, a 500 µL of phage volume at ≃10^9^ PFU/mL was introduced into a 1.5 mL centrifuge tube containing an equal volume of 12 µg/mL of propolis for 3 h. The stability of the phage was assessed by determining the phage titer before and after incubation with the propolis.

### Time-killing curve and one-step growth curve

The bacteriolytic activity was conducted with minor modifications, as described previously [76]. Briefly, the bacteriolytic efficiency of the phage at MOIs of 0.1, 1, and 10, was evaluated at various time points over period of 3.5 h of incubation. The experiment included two centrifuge tubes (C and BS), the C (control) tube contained only bacterial culture (not treated with phage), while the BS (bacterial survival) tube contained a bacterial culture treated with the phage. Each tube containing 10 mL of the exponential-growth-phase culture of EF/14 with a concentration adjusted (≃10^5^ CFU/mL). At each time point, an aliquot of 100 µL was collected and then incubated at 37 °C with shaking at 100 rpm. At each time point, an aliquot of 100 µL was collected, then serially diluted and spotted on TSA plates [79].

The one-step growth curve was conducted to estimate the infection latency and the burst size at MOI 0.1, from which the phage plaques were enumerated by collecting two aliquots, one for the infective centers (IC, untreated phage lysate with chloroform) and another chloroform-treated aliquot [76].

### Phage anti-biofilm efficacy

The phage's anti-biofilm efficacy was evaluated across various phage MOIs (0.001–100, equivalent to 10^4^ PFU/mL—10^9^ PFU/mL) using a crystal violet assay in a microtiter plate, as described by a previous study [80]. The antibiofilm activity was assessed regarding biofilm inhibition and clearance of preformed biofilm.

### Biofilm inhibition

In brief, a volume of 20 μL of phage was added to 180 μL of bacterial suspension (10^6^ CFU/mL) that was incubated in a 96-well plate (CELLSTAR, Greiner Bio-One, Portugal) at 37 °C overnight. The following day, the supernatant was removed, and the wells were washed with dH_2_O three times, then incubated for 1 h at 60 °C for heat fixation [81]. In addition, 150 µL of 0.06% (w/v) crystal violet was added to each well and incubated for 5 min in a dark place. After that, the crystal violet was removed and rewashed three times with dH_2_O, then the plate was left to dry. Following staining, the biofilm was eluted with 200 µL of 30% (v/v) glacial acetic acid and subsequent measurement at an optical density (OD) of 595 nm using the FLUOstar® Omega Microplate Reader (BMG LABTECH, Germany). Six replicates were used for each MOI evaluation.

#### Biofilm clearance

The standard biofilm inhibition assay was modified. Instead of immediate phage treatment, bacterial cultures were incubated for 24 h to establish the EF/14 biofilm. Subsequently, phage preparations at different MOIs were added to the untreated biofilm and incubated at 37°C for 24 h.

### Phage imaging using transmission electron microscopy (TEM)

The purified phage was used for phage morphology and imaged on a glow-discharged using TEM (1230 JEOL, Tokyo, Japan). Exactly 10 µL of high-titer phage was dropped onto a carbon-coated copper (Pelco International) grid for 2 min and stained with 4% uranyl acetate for phage visualization. The captured images from a JEOL 1230 TEM were measured using ImageJ software version 1.53n [82].

### DNA isolation and purification

The phenol–chloroform-isoamyl alcohol method was utilized, as previously described [83], to extract the phage genome from a high-titer phage lysate (10^9^ PFU/mL). Briefly, the phage was purified from bacteria using a syringe filter. Next, DNase and RNase were used to degrade free nucleic acids before breaking the phage capsid with proteinase K, sodium dodecyl sulfate (SDS), and ethylenediaminetetraacetic acid (EDTA, Prevest Denpro Limited Company, Jammu, India) at 56 °C for 1 h. An equal volume of phenol, chloroform, and isoamyl alcohol (25:24:1) was added to isolate genomic DNA. After centrifugation at 18,000×*g* for 10 min, the genomic DNA mixture was separated into a top layer of aqueous phase. The isolated DNA was precipitated by adding 3M sodium acetate and ice-cold isopropanol, then left for 1 h at − 80°C. The DNA pellet was formed by centrifugation at 18,000×*g* for 10 min. The pellet was washed twice in 90% and 70% ice-cold ethanol, then kept to dry before adding 100 μL DNase- and RNase-free water.

### Sequencing and bioinformatics analysis

The analysis of obtained reads was conducted using FastQC (v0.12.1) and de novo assembled with Unicycler (v0.4.8) on the BV-BRC platform [84]. The resultant single phage contig was annotated with the Rapid Annotation using Subsystem Technology Toolkit (RASTtk) pipeline [85–87]. The predicted ORFs were further checked for homology with protein sequences in the NCBI non-redundant protein database by BLASTp. Additionally, the ORF sequences were analyzed by InterProScan against InterPro's member database signatures. tRNAscan [88] and Aragon [89] were used to find tRNA genes in the phage genomic sequence.

The suitability of the phage for therapy was evaluated by analyzing its genome for genetic markers associated with antimicrobial resistance (AR), virulence, lysogeny, and diversity-generating retroelements (DGR) systems. This analysis was performed using PhageLeads [90], the Resistance Gene Identifier (RGI) on the Comprehensive Antibiotic Resistance Database (CARD) [91], and myDGR server [92]. The genome map of phage vB_EfaS_ZC1 was visualized using Proksee [93]. Phage coding sequences were screened for depolymerase domains using DPO [94], and the topologies of the predicted amino acid sequences were analyzed for transmembrane domains with DeepTMHMM [95].

Genome-genome distancing was assessed by ViPtree (proteome comparison) [96] and VIRIDIC (nucleotide sequence comparison) [97]. VIRIDIC assigned taxa using ICTV species and genus thresholds. Additionally, the closest-related phages to vB_EfaS_ZC1 were analyzed for the homology of their large subunit terminase (TerL), a core protein, and a phylogenetic tree was constructed on Phylogeny.fr [98]. To infer evolutionary relationships, amino acid sequences were aligned using MUSCLE (v3.8.31) [99], followed by gap removal with Gblocks (v0.91b). The WAG substitution model was selected and incorporated into the PhyML program (v3.1/3.0 aLRT) for maximum likelihood phylogenetic tree reconstruction [100]. Branch reliability within the tree was assessed using the aLRT (SH-Like) test [101]. Finally, TreeDyn (v198.3) was used to visualize the phylogenetic tree [102].

### Propolis extraction

The ethanol propolis extraction method was prepared according to the previous study with some modifications [103]. Initially, 4 g of the raw propolis from a bee farm in Siwa Oasis, Egypt, was added to 100 mL of 80% ethanol; the extract was kept at 80 °C for 2 h after being incubated at 60 °C for the same time, followed by centrifugation at high speed to precipitate large particles. The last step was collection and purification using a syringe filter for the supernatant, and then stored at − 20 °C.

### Disc and well diffusion methods

Ramadan et al. [104] intended to assess the antibacterial efficacy of propolis against EF/14 host strain using the agar well and disc diffusion technique. A volume of 100 μL of bacterial culture of EF/14 (10^8^ CFU/mL) was spread with sterilized cotton on TSA plates. After the upper inoculated agar medium was dried, various wells were created using sterile tips. For the disc diffusion, sterile filter paper discs with a diameter of approximately 6 mm were positioned onto the surface of TSA agar. Subsequently, 10 µl of propolis was added at various concentrations ranging from 6.25 to 100 µg/mL, and with sterilized broth as a negative control.

### Minimum inhibitory concentration (MIC) and minimum bactericidal concentration (MBC) of propolis

MIC and MBC determinations for the propolis extract were conducted as previously reported [105]. Briefly, serial dilutions of propolis (6.25–100 µg/mL) were prepared in a 96-well plate and inoculated with EF/14 (10^6^ CFU/mL). After 24 h of incubation at 37 °C, the MIC was determined as the lowest extract concentration preventing visible growth. To assess the MBC, cultures from clear wells were serially diluted and plated on TSA for colony counting.

### Preparation of *E. faecalis* phage combined with propolis

#### Time-killing curve assay of combination

The bactericidal effects of the phage, propolis, and their combination (phage-propolis) against EF/14 were determined by measuring the CFU/mL in triplicates. The experiment was divided into four groups: phage 10^8^ PFU/mL, propolis 12.5 µg/mL, combination (phage 10^8^ PFU/mL, propolis 12.5 µg/mL), and bacterial culture (10^7^ CFU/mL) at MOI ≃1. Each group was prepared with a final volume of 6 mL and then incubated at shaking incubator at 37 °C. Aliquots of 100 µL in each tube were taken from each tube at intervals of 0 min, 30 min, 60 min, 90 min, 120 min, 180 min, and 48 h. These samples were subjected to tenfold serial dilution and then spotted on TSA plates, followed by incubation at 37 °C for 24 h [106].

### Application of *E. faecalis* phage therapy ex vivo human tooth infected dentin models

#### Preparation of the ex vivo human tooth-infected dentin model

##### Sample selection

Eighty-one single-rooted, human mandibular second premolar teeth that were freshly extracted for orthodontics or prosthetic causes were collected from an outpatient clinic, Faculty of Dentistry, Suez Canal University, under protocol approval by the faculty ethics committee (No. 703/2023). The teeth were preoperatively evaluated by digital periapical radiographs in buccolingual and mesiodistal dimensions, following inclusion criteria: sound single root with single canal type I in Vertucci’s classification [107], and closed apices. The following teeth were excluded if they had previous endodontic manipulation, apical ramifications, root carious lesions, fractures or cracks, hyper-cementosis, root canal calcifications, or resorption. The sample size was calculated according to Faul et al*.* [108] using G*Power software 3.1.9.6 (G Power; Franz Faul, University of Kiel, Germany, Supplementary Figures S1 and S2).

##### Root Slice Preparation

The teeth were subjected to an ultrasonic scaler (Miltex, Davies Drive, York) to eliminate debris and calculus, followed by a 10 min ultrasonic rinse in distilled water (ddH_2_O). Then, the tooth slices were immersed in 5.25% NaOCl for 30 min. A final wash using a 5% sodium thiosulfate solution (Na_2_S_2_O_3_) was performed To neutralize the NaOCl [109]. Teeth were decoronated below the cementoenamel junction to a standardized length of 16 mm using a rotary diamond disk (Sharp Inc, Luzern, Switzerland) attached to a low-speed handpiece with water coolant. Each tooth's canal patency and working length were checked radiographically using k files of size #15. All root canals were then prepared by using E-FLEX Gold heat-treated nickel-titanium rotary files using an E-connect endo motor (Eighteeth Medical, Changzhou City, Jiangsu Province, China) in a rotation mode, set at 350 rpm and 2.5 Ncm torque using step-down technique according to manufacturer's instructions until the master apical file 35/06 was reached. Three milliliters of 3% NaOCl were irrigated in each root canal for 3 min using a 3 mL plastic syringe with a 30-gauge side-vented needle (Laigues, Vd, Switzerland). Subsequently, the canals were irrigated with 3 mL of 17% EDTA for 5 min to eliminate the smear layer. To neutralize any residual EDTA or NaOCl, the canals were rinsed with 10 mL of 10% ddH_2_O followed by 5 mL of 5% Na_2_S_2_O_3_. The external surfaces of the prepared root samples were then coated with two layers of nail polish.

The prepared roots were split longitudinally into two halves and then horizontally to obtain standardized root segments of five mm in length, creating a total of 162 semi-circular root samples (*n* = 162). Then, all tooth slices were sterilized in an autoclave at 121 °C for 20 min in TSB broth. Additionally, one tooth slice from each group (n = 1) was randomly selected for FE-SEM evaluation to confirm the sterilization of the samples.

### Biofilm-infected root dentin slices

#### Cultivation and inoculation of biofilm

*E. faecalis* was cultured on TSA agar at 37 °C for 24 h, then the colonies were suspended in 20 mL of TSB. Each sterilized tooth slice was placed on a 24-well cell culture plate facing upward. After that, each tooth slice was inoculated with *E. faecalis* suspension with a turbidity of (1.5 × 10^8^ CFU/mL) using a sterile insulin syringe (AdvaCare, Cheyenne, USA). The infected tooth slices were incubated for 4 weeks at 37 °C under aseptic aerobic conditions to allow biofilm formation. Then, it is replenished with fresh TSB broth every 3 days to ensure bacterial viability and prevent death [58, 110].

##### Evaluation of biofilm formation at 4 weeks

Biofilm formation was measured from one random sample from each group (n = 1) after 28 days using FE-SEM. This process ensured that all samples had been infected successfully with mature bacterial biofilm before testing the irrigation treatments.

##### Application of irrigation treatment on infected dentin slices

By the end of the 4 weeks, samples were washed using 5 mL of Phosphate Buffered Saline (PBS) to eliminate the remaining broth. Afterward, each of the remaining prepared tooth slices (n = 25) in each group was immersed in 1 mL of corresponding tested irrigation contained in a centrifuge tube for 10 min [111, 112]. The irrigant was applied as follows:

- Group A: No treatment (serves as a blank control)
- Group B: four-week mature biofilm without any treatment (serves as a negative control)
- Group C: 2% NaOCl (serves as a positive control)
- Group D: 0.9% Saline
- Group E: 12 µg/mL Propolis
- Group F: 10^8^ PFU/mL Phage
- Group G: A combination of 10^8^ PFU/mL Phage + 12 µg/mL Propolis

#### Evaluation of biofilm treatment on dentin slices with different test irrigants

##### Viable bacterial count assessment after irrigation

After treating the root slices with corresponding irrigation, each specimen was positioned in a separate petri dish. The dentinal shavings were collected into a centrifuge tube containing 1 mL of TSB. Exactly 100 µL of media with dentinal shavings were added to 900 µL sterile TSB then, serially diluted and spotted onto TSA agar plates.

### FE-SEM qualitative analysis

After irrigation, tooth slices were washed with 0.1% Na_2_S_2_O_3,_ followed by PBS for 1 min to neutralize hypochlorite and remove the remaining irrigation solution. All specimens were sputter-coated with gold surface roughness (Hummer 8, for 3 min, 15 milliamperes) using carbon paste on a copper stub. The sputter-coated tooth slices were examined using FE-SEM (ZEISS, LEO SUPRA-55, Germany) at various magnifications (1,000X, 1,500X, 2,000X, and 4,000X) to assess biofilm formation on the surface qualitatively. This approach allowed for a detailed comparison of biofilm morphology between the different irrigation groups.

### CLSM quantitative analysis

Following treatment removal, the tooth slices were rinsed with 0.1% Na_2_S_2_O_3,_ followed by PBS for 1 min, then left to dry. The samples were stained with fluorescent LIVE/DEAD BacLight bacterial viability stain, Acridine Orange (AO), and propidium iodide (PI) for 15 min. Live and dead cells were visualized using green and red fluorescence, respectively. Subsequently, the sample was examined under CLSM (Zeiss, LSM 980, Jena, Germany) with × 50 magnification. Zeiss Zen 3.2 (blue edition) software was utilized to analyze and quantify bacterial cells using CLSM images to ensure precise and accurate results.

### Data analysis

All statistical analysis was conducted using SPSS software (version 20, SPSS Inc., USA) and GraphPad Prism 9.0 software (San Diego, CA, USA). Comparison between control and test groups was performed using one-way ANOVA, and Tukey's post-hoc test with a significance threshold set at *P* < 0.05.

### Accession number

The annotated genome of *Enterococcus* phage vB_EfaS_ZC1 has accession number PP271740 and taxon ID 311956.

## Supplementary Information

Additional file 1.

## Data Availability

All data supporting the findings of this study are available within the article and its supplementary materials. The phage vB_EfaS_ZC1 is publicly accessible with an accession number PP271740 and taxon ID 311956 on NCBI.

## Abbreviations

AO: Acridine orange
AR: Antimicrobial resistance
BS: Bacterial survival
C: Control
CAPE: Caffeic acid phenethyl ester
CARD: Comprehensive antibiotic resistance database
CFU: Colony-forming unit
CLSM: Confocal laser scanning microscopy
ddH_2_O: Distilled water
DT: Dentinal tubules
DGR: Diversity-generating retroelements
EDTA: Ethylenediaminetetraacetic acid
FE-SEM: Field emission scanning electron microscopy
IC: Infective centers
MBC: Minimum Bactericidal Concentration
MDR: Multidrug-Resistant
MIC: Minimum Inhibitory Concentration
MOI: Multiplicities of infection
NaOCl: Sodium hypochlorite
Na_2_S_2_O_3_: Sodium thiosulfate solution
OD: Optical density
ORF: Open reading frame
PBS: Phosphate buffered saline
PFU: Plaque-forming unit
PI: Propidium iodide
RASTtk: Rapid annotation using subsystem technology toolkit
RGI: Resistance gene identifier
SDS: Sodium dodecyl sulfate
TerL: Large subunit terminase
VIRIDIC: Virus intergenomis distance calculator
VREF: Vancomycin-resistant *E. faecalis*
