# Supplementary material for: Novel strategies for vancomycin-resistant Enterococcus faecalis biofilm control: bacteriophage (vB_EfaS_ZC1), propolis, and their combined effects in an ex vivo endodontic model
Source: Ann Clin Microbiol Antimicrob. 2025 Apr 13;24:24. doi: 10.1186/s12941-025-00790-y (PMC11995525; doi:10.1186/s12941-025-00790-y)
Supplement: Supplementary file 1 — Additional file 1. [file 12941_2025_790_MOESM1_ESM.docx]

1. **Bacterial Confirmation and Identification of Virulence Genes by Polymerase Chain Reaction (PCR)**

The PCR mixture included bacterial suspension, primers, HotStarTaq master mixture, and MgCl2. The amplification protocol involved initial activation to activate the HotStarTaq DNA polymerase, followed by 30 cycles of denaturation, annealing, and extension. After amplification, 25 μl of the amplicon was mixed with 5 μl of gel loading buffer (50% glycerol, 0.8 mg of bromophenol blue per mL) and electrophoresed in a 1.5% (W/V) agarose gel (SphaeroQ, Burgos, Spain) for 1 h at 150 V in 1× TBE (Tris-borate-EDTA) containing 5 μl of ethidium. A 100-bp DNA ladder (Invitrogen, Merelbeke, Belgium) was used as a molecular size marker. Finally, the following combinations of PCR reactions were standardized [40, 41]. All the primers used in the current study were purchased from Eurofins.

**Table S1. The target genes, primer sequences, annealing temperature, and product size of the PCR reaction used in the study.**

| **Primers** | **Oligonucleotide primer sequences (5'-3')** | **Annealing Temperature** | **Product size (bp)** | **Ref.** |
| --- | --- | --- | --- | --- |
| *ddl* *_E. faecalis_* | F: 5’-ATCAAGTACAGTTAGTCT-3’  R: 5’-ACGATTCAAAGCTAACTG-3’ | 57°C | 941bp | [2] |
| *esp*  (Enterococcal surface protein) | F: 5’-AGATTTCATCTTTGATTCTTGG-3’  R: 5’-AATTGATTCTTTAGCATCTGG-3’ | 56°C | 510 bp | [3] |
| *gelE*  (Gelatinase) | F: 5’-TATGACAATGCTTTTTGGGAT-3’  R: 5’- AGATGCACCCGAAATAATATA-3’ | 56°C | 213 bp | [4] |
| *asa1* (Aggregation substance) | F: 5’-GCACGCTATTACGAACTATGA-3’  R: 5’-TAAGAAAGAACATCACCACGA-3’ | 56°C | 375 bp | [5] |
| *cylA*  (Cytolysin) | F: 5’-ACTCGGGGATTGATAGGC-3’  R: 5’-GCTGCTAAAGCTGCGCTT-3’ | 56°C | 688 bp | [6] |
| *Hyl*  (Hyaluronidase) | F: 5’- ACAGAAGAGCTGCAGGAAATG-3’  R: 5’- GACTGACGTCCAAGTTTCCAA-3’ | 56°C | 276 bp | [7] |

1. **Preparation of the *ex vivo* human tooth-infected dentine model**
   1. **Sample size calculation.**

The sample size was calculated according to Faul et al. [1] using G*Power software 3.1.9.6 (G Power; Franz Faul, University of Kiel, Germany). The minimum calculated total sample size was 162 sufficient to detect an effect size of 0.23 (Small) and a power (1-β=0.95) of 95%, at a significance probability level of p<0.05. According to sample size calculations, a total of 150 root slices were used during the current study, 60 slices for CFU which was divided into 6 groups (10 root samples per group), 60 samples for CLSM (10 per group), and 30 slices for SEM analysis; were divided into 6 groups (5 per group), so each experimental group contains 25 root slices. Additional two root slice samples were added to each group, one for SEM validation of sample disinfection and the other one sample for biofilm formation confirmation in each group before the application of tested irrigants. So, the total number of full roots used was 81 full roots to obtain 162 root slices, to use 27 root slices in each group (n=27).”

**Figure S1. The current study's sample size graph was generated by G*Power software. This graph depicts the relationships between sample size, statistical power, and effect size.**

**Figure S2. The relationships between the sample size needed, statistical power (1-β), anticipated effect size, and the chosen significance level (α) for the current study.**

**Table S2. Annotated CDS of phage vB_EFaS_ZC1 (Accession number: PP271740)**

| **CDS #** | **Position (nt)** | | **Length bp** | **Strand** | **Product** | **Functional Category** |
| --- | --- | --- | --- | --- | --- | --- |
|  | **Start** | **Stop** |  |  |  |  |
| **ORF1** | 402 | 262 | 141 | - | hypothetical protein | Hypothetical protein |
| **ORF2** | 791 | 579 | 213 | - | hypothetical protein | Hypothetical protein |
| **ORF3** | 1011 | 793 | 219 | - | hypothetical protein | Hypothetical protein |
| **ORF4** | 1800 | 1012 | 789 | - | hypothetical protein | Hypothetical protein |
| **ORF5** | 2622 | 2251 | 372 | - | hypothetical protein | Hypothetical protein |
| **ORF6** | 2944 | 2642 | 303 | - | hypothetical protein | Hypothetical protein |
| **ORF7** | 3209 | 2946 | 264 | - | Thioredoxin | Unsorted |
| **ORF8** | 3371 | 3234 | 138 | - | hypothetical protein | Hypothetical protein |
| **ORF9** | 3755 | 3375 | 381 | - | Protein with methytransferase activity | Immune |
| **ORF10** | 4146 | 3757 | 390 | - | hypothetical protein | Hypothetical protein |
| **ORF11** | 4403 | 4146 | 258 | - | hypothetical protein | Hypothetical protein |
| **ORF12** | 4717 | 4403 | 315 | - | hypothetical protein | Hypothetical protein |
| **ORF13** | 4998 | 5597 | 600 | + | Terminase, small subunit | Packaging |
| **ORF14** | 5611 | 5955 | 345 | + | hypothetical protein | Hypothetical protein |
| **ORF15** | 5974 | 6222 | 249 | + | Holin | Lysis |
| **ORF16** | 6285 | 7556 | 1272 | + | Terminase, large subunit | Packaging |
| **ORF17** | 7613 | 9148 | 1536 | + | Portal protein | Assembly |
| **ORF18** | 9160 | 9915 | 756 | + | Head morphogenesis protein | Assembly |
| **ORF19** | 10026 | 10700 | 675 | + | Head scaffolding protein | Assembly |
| **ORF20** | 10749 | 11555 | 807 | + | Major capsid protein | Assembly |
| **ORF21** | 11710 | 12150 | 441 | + | Major tail protein | Assembly |
| **ORF22** | 12210 | 12614 | 405 | + | Phage head-to-tail connector protein | Assembly |
| **ORF23** | 12630 | 13007 | 378 | + | hypothetical protein | Hypothetical protein |
| **ORF24** | 12992 | 13372 | 381 | + | hypothetical protein | Hypothetical protein |
| **ORF25** | 13385 | 13819 | 435 | + | Phage tail-to-head joining protein | Assembly |
| **ORF26** | 13840 | 14529 | 690 | + | Major tail protein | Assembly |
| **ORF27** | 14673 | 15113 | 441 | + | hypothetical protein | Hypothetical protein |
| **ORF28** | 15124 | 15348 | 225 | + | hypothetical protein | Hypothetical protein |
| **ORF29** | 15361 | 18246 | 2886 | + | Phage tail tape measure protein | Assembly |
| **ORF30** | 18260 | 22252 | 3993 | + | Phage tail fiber protein with depolymerase domain | Assembly and Infection |
| **ORF31** | 22265 | 25291 | 3027 | + | Phage tail spike with depolymerase domain | Assembly and Infection |
| **ORF32** | 25305 | 25592 | 288 | + | hypothetical protein | Hypothetical protein |
| **ORF33** | 25639 | 26352 | 714 | + | N-acetylmuramoyl-L-alanine amidase (Endolysin) | Lysis |
| **ORF34** | 27108 | 26635 | 474 | - | hypothetical protein | Hypothetical protein |
| **ORF35** | 27132 | 27329 | 198 | + | hypothetical protein | Hypothetical protein |
| **ORF36** | 27566 | 28390 | 825 | + | hypothetical protein | Hypothetical protein |
| **ORF37** | 28443 | 28877 | 435 | + | hypothetical protein | Hypothetical protein |
| **ORF38** | 28880 | 29275 | 396 | + | hypothetical protein | Hypothetical protein |
| **ORF39** | 29265 | 29465 | 201 | + | hypothetical protein | Hypothetical protein |
| **trna1** | 29703 | 29774 | 72 | + | tRNA-Trp-CCA | tRNA |
| **trna2** | 29777 | 29865 | 89 | + | tRNA-Ser-TGA | tRNA |
| **ORF40** | 29894 | 30019 | 126 | + | hypothetical protein | Hypothetical protein |
| **ORF41** | 30816 | 31157 | 342 | + | hypothetical protein | Hypothetical protein |
| **ORF42** | 31157 | 31309 | 153 | + | hypothetical protein | Hypothetical protein |
| **ORF43** | 31340 | 31591 | 252 | + | hypothetical protein | Hypothetical protein |
| **ORF44** | 31591 | 31743 | 153 | + | hypothetical protein | Hypothetical protein |
| **ORF45** | 31757 | 31945 | 189 | + | hypothetical protein | Hypothetical protein |
| **ORF46** | 31957 | 32169 | 213 | + | hypothetical protein | Hypothetical protein |
| **ORF47** | 32199 | 33143 | 945 | + | DNA primase | Replication |
| **ORF48** | 33218 | 33571 | 354 | + | Transcriptional regulator | Regulation |
| **ORF49** | 33620 | 34396 | 777 | + | DNA replication protein | Replication |
| **ORF50** | 34408 | 35772 | 1365 | + | Replicative DNA helicase | Replication |
| **ORF51** | 35785 | 36537 | 753 | + | DNA methylase | Immune |
| **ORF52** | 36503 | 36727 | 225 | + | hypothetical protein | Hypothetical protein |
| **ORF53** | 36739 | 36999 | 261 | + | hypothetical protein | Hypothetical protein |
| **ORF54** | 37072 | 37524 | 453 | + | HNH endonuclease | Packaging |
| **ORF55** | 37509 | 38537 | 1029 | + | Exonuclease | Unsorted |
| **ORF56** | 38537 | 38872 | 336 | + | hypothetical protein | Hypothetical protein |
| **ORF57** | 38869 | 39438 | 570 | + | Crossover junction endodeoxyribonuclease RuvC | Regulation |
| **ORF58** | 39435 | 40004 | 570 | + | Putative adenylate kinase | Unsorted |
| **ORF59** | 39997 | 40629 | 633 | + | Winged helix-like DNA-binding domain | Regulation |
| **ORF60** | 40721 | 41284 | 564 | + | LPS glycosyltransferase | Hypothetical protein |
| **ORF61** | 41287 | 41403 | 117 | + | hypothetical protein | Immune |
| **ORF62** | 41415 | 41588 | 174 | + | hypothetical protein | Hypothetical protein |
| **ORF63** | 41603 | 41782 | 180 | + | hypothetical protein | Hypothetical protein |
| **ORF64** | 41783 | 41977 | 195 | + | hypothetical protein | Hypothetical protein |
| **ORF65** | 41992 | 42213 | 222 | + | hypothetical protein | Hypothetical protein |
| **ORF66** | 42213 | 42422 | 210 | + | hypothetical protein | Hypothetical protein |
| **ORF67** | 42425 | 42637 | 213 | + | hypothetical protein | Hypothetical protein |
| **ORF68** | 42638 | 42991 | 354 | + | hypothetical protein | Hypothetical protein |
| **ORF69** | 42991 | 43176 | 186 | + | hypothetical protein | Hypothetical protein |
| **ORF70** | 43255 | 45609 | 2355 | + | DNA polymerase I | Hypothetical protein |
| **ORF71** | 45602 | 46138 | 537 | + | HNH nuclease | Replication |
| **ORF72** | 46222 | 46656 | 435 | + | hypothetical protein | Packaging |
| **ORF73** | 46658 | 47050 | 393 | + | hypothetical protein | Hypothetical protein |
| **ORF74** | 47043 | 47738 | 696 | + | ATP-dependent protease | Hypothetical protein |
| **ORF75** | 47797 | 48201 | 405 | + | hypothetical protein | Lysis |
| **ORF76** | 48185 | 48421 | 237 | + | hypothetical protein | Hypothetical protein |
| **ORF77** | 48418 | 48714 | 297 | + | hypothetical protein | Hypothetical protein |
| **ORF78** | 48737 | 49321 | 585 | + | hypothetical protein | Hypothetical protein |
| **ORF79** | 49332 | 49532 | 201 | + | hypothetical protein | Hypothetical protein |
| **ORF80** | 49545 | 49976 | 432 | + | hypothetical protein | Hypothetical protein |
| **ORF81** | 50055 | 50249 | 195 | + | hypothetical protein | Hypothetical protein |
| **ORF82** | 50246 | 50506 | 261 | + | hypothetical protein | Hypothetical protein |
| **ORF83** | 50506 | 50718 | 213 | + | hypothetical protein | Hypothetical protein |
| **ORF84** | 50731 | 50949 | 219 | + | hypothetical protein | Hypothetical protein |
| **ORF85** | 50939 | 51148 | 210 | + | hypothetical protein | Hypothetical protein |
| **ORF86** | 51162 | 51410 | 249 | + | hypothetical protein | Hypothetical protein |
| **ORF87** | 51403 | 51669 | 267 | + | hypothetical protein | Hypothetical protein |
| **ORF88** | 51671 | 52633 | 963 | + | RNA ligase | Hypothetical protein |
| **ORF89** | 52680 | 52985 | 306 | + | Phosphoribosyl-ATP pyrophosphohydrolase-like | Replication |
| **ORF90** | 52988 | 53500 | 513 | + | hypothetical protein | Replication |
| **ORF91** | 54251 | 54574 | 324 | + | hypothetical protein | Hypothetical protein |
| **ORF92** | 54567 | 54671 | 105 | + | hypothetical protein | Hypothetical protein |
| **ORF93** | 54673 | 55059 | 387 | + | hypothetical protein | Hypothetical protein |
| **ORF94** | 55052 | 55426 | 375 | + | hypothetical protein | Hypothetical protein |
| **ORF95** | 55504 | 55638 | 135 | + | hypothetical protein | Hypothetical protein |
| **ORF96** | 55776 | 56210 | 435 | + | hypothetical protein | Hypothetical protein |
| **ORF97** | 56281 | 56406 | 126 | + | hypothetical protein | Hypothetical protein |
| **ORF98** | 56487 | 56807 | 321 | + | hypothetical protein | Hypothetical protein |
| **ORF99** | 56941 | 57189 | 249 | + | hypothetical protein | Hypothetical protein |
| **ORF100** | 57269 | 57415 | 147 | + | hypothetical protein | Hypothetical protein |
| **ORF101** | 57506 | 57808 | 303 | + | hypothetical protein | Hypothetical protein |
| **ORF102** | 57925 | 58038 | 114 | + | hypothetical protein | Hypothetical protein |

**
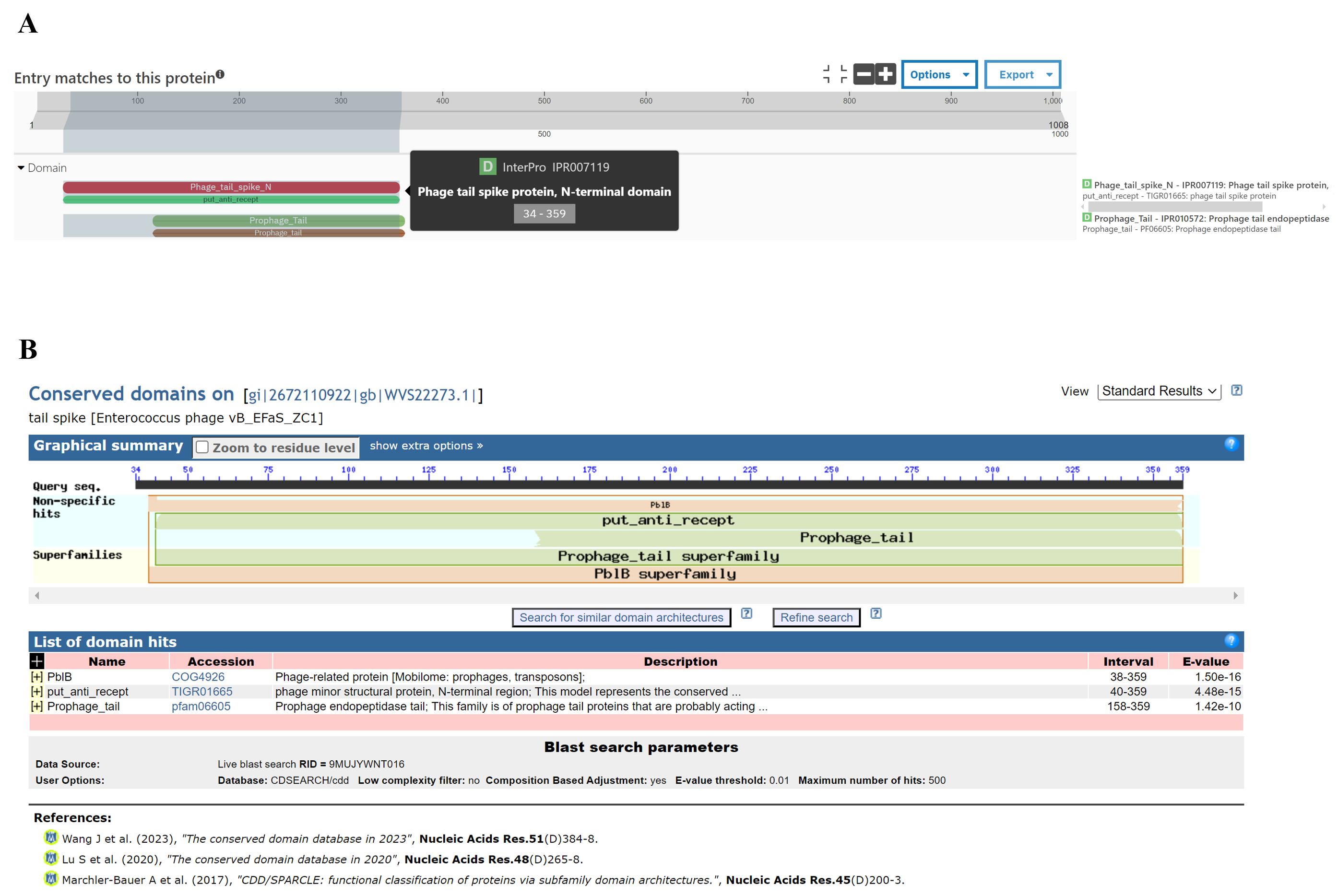
**

**Figure S3. InterProScan (A) and UniProt BLAST (B) analyses of the putative tail spike protein (ORF31).**

**Table S3. Description of BLASTp results of N-terminal domain (34-** **359 aa) of tail spike protein against deposited protein sequences of saphexaviruses on NCBI database.**

| **Description** | **Max Score** | **Total Score** | **Query Cover** | **E value** | **Per. ident** | **Accession** |
| --- | --- | --- | --- | --- | --- | --- |
| tail spike [Enterococcus phage vB_EFaS_ZC1] | 685 | 685 | 100% | 0 | 100 | [WVS22273.1](https://www.ncbi.nlm.nih.gov/protein/WVS22273.1?report=genbank&log$=prottop&blast_rank=3&RID=9YRNT7GX016) |
| tail spike protein [Enterococcus phage vB_EfKS5] | 685 | 685 | 100% | 0 | 100 | [WDS60755.1](https://www.ncbi.nlm.nih.gov/protein/WDS60755.1?report=genbank&log$=prottop&blast_rank=1&RID=9YRNT7GX016) |
| tail assembly [Enterococcus phage vB_OCPT_SDS2] | 685 | 685 | 100% | 0 | 100 | [UQT00917.1](https://www.ncbi.nlm.nih.gov/protein/UQT00917.1?report=genbank&log$=prottop&blast_rank=2&RID=9YRNT7GX016) |
| putative phage minor structural protein [Enterococcus phage phiM1EF2] | 684 | 684 | 100% | 0 | 100 | [BCF74639.1](https://www.ncbi.nlm.nih.gov/protein/BCF74639.1?report=genbank&log$=prottop&blast_rank=4&RID=9YRNT7GX016) |
| tail tip protein Tal [Enterococcus phage vB_Efs6_KEN03] | 684 | 684 | 100% | 0 | 100 | [WZP34684.1](https://www.ncbi.nlm.nih.gov/protein/WZP34684.1?report=genbank&log$=prottop&blast_rank=5&RID=9YRNT7GX016) |
| hyaluronidase [Enterococcus phage vB_EfaS_EF1c55] | 684 | 684 | 100% | 0 | 100 | [QEM41697.1](https://www.ncbi.nlm.nih.gov/protein/QEM41697.1?report=genbank&log$=prottop&blast_rank=6&RID=9YRNT7GX016) |
| virion structural protein [Enterococcus phage VD13] | 683 | 683 | 100% | 0 | 99.69 | [YP_009036392.1](https://www.ncbi.nlm.nih.gov/protein/YP_009036392.1?report=genbank&log$=prottop&blast_rank=7&RID=9YRNT7GX016) |
| minor structural protein [Enterococcus phage UTI-EfS7] | 678 | 678 | 100% | 0 | 98.77 | [UKM17594.1](https://www.ncbi.nlm.nih.gov/protein/UKM17594.1?report=genbank&log$=prottop&blast_rank=8&RID=9YRNT7GX016) |
| minor structural protein [Enterococcus phage PEF7b] | 675 | 675 | 100% | 0 | 98.47 | [WLG15335.1](https://www.ncbi.nlm.nih.gov/protein/WLG15335.1?report=genbank&log$=prottop&blast_rank=9&RID=9YRNT7GX016) |
| virion structural protein [Enterococcus phage IMEEF1] | 671 | 671 | 100% | 0 | 97.85 | [YP_009603972.1](https://www.ncbi.nlm.nih.gov/protein/YP_009603972.1?report=genbank&log$=prottop&blast_rank=10&RID=9YRNT7GX016) |
| minor structural protein [Enterococcus phage PEF9] | 671 | 671 | 100% | 0 | 97.85 | [WLG15414.1](https://www.ncbi.nlm.nih.gov/protein/WLG15414.1?report=genbank&log$=prottop&blast_rank=11&RID=9YRNT7GX016) |
| hypothetical protein KMDAMLD_00066 [Enterococcus phage vB_OCPT_PG11] | 671 | 671 | 100% | 0 | 97.55 | [UQT01537.1](https://www.ncbi.nlm.nih.gov/protein/UQT01537.1?report=genbank&log$=prottop&blast_rank=12&RID=9YRNT7GX016) |
| hypothetical protein LMOIWNZ_00012 [Enterococcus phage vB_OCPT_CCS3] | 671 | 671 | 100% | 0 | 97.55 | [UQT01038.1](https://www.ncbi.nlm.nih.gov/protein/UQT01038.1?report=genbank&log$=prottop&blast_rank=13&RID=9YRNT7GX016) |
| virion structural protein [Streptococcus phage SP-QS1] | 670 | 670 | 100% | 0 | 97.55 | [YP_008320497.1](https://www.ncbi.nlm.nih.gov/protein/YP_008320497.1?report=genbank&log$=prottop&blast_rank=14&RID=9YRNT7GX016) |
| hypothetical protein EMSIMAW_00060 [Enterococcus phage vB_OCPT_PG2] | 670 | 670 | 100% | 0 | 97.55 | [UQT01342.1](https://www.ncbi.nlm.nih.gov/protein/UQT01342.1?report=genbank&log$=prottop&blast_rank=15&RID=9YRNT7GX016) |
| minor structural protein [Enterococcus phage Toszka] | 670 | 670 | 100% | 0 | 97.55 | [WYA79031.1](https://www.ncbi.nlm.nih.gov/protein/WYA79031.1?report=genbank&log$=prottop&blast_rank=16&RID=9YRNT7GX016) |
| minor structural protein [Enterococcus phage vB_OCPT_SDS1] | 669 | 669 | 100% | 0 | 97.55 | [UQT00817.1](https://www.ncbi.nlm.nih.gov/protein/UQT00817.1?report=genbank&log$=prottop&blast_rank=17&RID=9YRNT7GX016) |
| tail spike protein [Enterococcus phage EF653P1] | 669 | 669 | 100% | 0 | 97.55 | [WAX15251.1](https://www.ncbi.nlm.nih.gov/protein/WAX15251.1?report=genbank&log$=prottop&blast_rank=18&RID=9YRNT7GX016) |
| minor structural protein [Enterococcus phage EfsWh-1] | 669 | 669 | 100% | 0 | 97.55 | [QAY01514.1](https://www.ncbi.nlm.nih.gov/protein/QAY01514.1?report=genbank&log$=prottop&blast_rank=19&RID=9YRNT7GX016) |
| hypothetical protein WMKKAML_00088 [Enterococcus phage vB_OCPT_PG9] | 669 | 669 | 100% | 0 | 97.55 | [UQT01465.1](https://www.ncbi.nlm.nih.gov/protein/UQT01465.1?report=genbank&log$=prottop&blast_rank=20&RID=9YRNT7GX016) |
| hypothetical protein QOFMPA_00079 [Enterococcus phage vB_OCPT_Toy] | 669 | 669 | 100% | 0 | 97.55 | [UQT01202.1](https://www.ncbi.nlm.nih.gov/protein/UQT01202.1?report=genbank&log$=prottop&blast_rank=21&RID=9YRNT7GX016) |
| hypothetical protein XMKAXML_00039 [Enterococcus phage vB_OCPT_PG13] | 668 | 668 | 100% | 0 | 97.24 | [UQT01605.1](https://www.ncbi.nlm.nih.gov/protein/UQT01605.1?report=genbank&log$=prottop&blast_rank=22&RID=9YRNT7GX016) |
| minor structural protein [Enterococcus phage EFap05-1] | 668 | 668 | 100% | 0 | 96.93 | [UIE13796.1](https://www.ncbi.nlm.nih.gov/protein/UIE13796.1?report=genbank&log$=prottop&blast_rank=23&RID=9YRNT7GX016) |
| minor structural protein [Enterococcus phage vB_EfaS_TV16] | 668 | 668 | 100% | 0 | 97.24 | [QIG60320.1](https://www.ncbi.nlm.nih.gov/protein/QIG60320.1?report=genbank&log$=prottop&blast_rank=24&RID=9YRNT7GX016) |
| minor structural protein [Enterococcus phage Entf1] | 668 | 668 | 100% | 0 | 96.93 | [QDB70490.1](https://www.ncbi.nlm.nih.gov/protein/QDB70490.1?report=genbank&log$=prottop&blast_rank=25&RID=9YRNT7GX016) |
| hypothetical protein vBEfaSHEf13_054 [Enterococcus phage vB_EfaS_HEf13] | 668 | 668 | 100% | 0 | 97.24 | [AYH92710.1](https://www.ncbi.nlm.nih.gov/protein/AYH92710.1?report=genbank&log$=prottop&blast_rank=26&RID=9YRNT7GX016) |
| tape measure protein [Enterococcus phage SSsP-1] | 668 | 668 | 100% | 0 | 97.24 | [QYI86595.1](https://www.ncbi.nlm.nih.gov/protein/QYI86595.1?report=genbank&log$=prottop&blast_rank=27&RID=9YRNT7GX016) |
| virion structural protein [Enterococcus phage EFKL] | 665 | 665 | 100% | 0 | 96.63 | [WAK45078.1](https://www.ncbi.nlm.nih.gov/protein/WAK45078.1?report=genbank&log$=prottop&blast_rank=28&RID=9YRNT7GX016) |
| virion structural protein [Enterococcus phage vB_EfaS_IME198] | 665 | 665 | 100% | 0 | 96.63 | [YP_009218896.1](https://www.ncbi.nlm.nih.gov/protein/YP_009218896.1?report=genbank&log$=prottop&blast_rank=29&RID=9YRNT7GX016) |
| tail tip protein Tal [Enterococcus phage vB_Efs19_KEN17] | 663 | 663 | 100% | 0 | 96.32 | [WZP35856.1](https://www.ncbi.nlm.nih.gov/protein/WZP35856.1?report=genbank&log$=prottop&blast_rank=30&RID=9YRNT7GX016) |
| minor structural protein [Enterococcus phage EF-P29] | 663 | 663 | 100% | 0 | 96.32 | [APU00250.1](https://www.ncbi.nlm.nih.gov/protein/APU00250.1?report=genbank&log$=prottop&blast_rank=31&RID=9YRNT7GX016) |
| virion structural protein [Enterococcus phage EF_KTM] | 660 | 660 | 100% | 0 | 96.93 | [WVP83319.1](https://www.ncbi.nlm.nih.gov/protein/WVP83319.1?report=genbank&log$=prottop&blast_rank=32&RID=9YRNT7GX016) |
| virion structural protein [Enterococcus phage EF_FB] | 659 | 659 | 100% | 0 | 96.63 | [WVP83163.1](https://www.ncbi.nlm.nih.gov/protein/WVP83163.1?report=genbank&log$=prottop&blast_rank=33&RID=9YRNT7GX016) |
| virion structural protein [Enterococcus phage EF_CM] | 656 | 656 | 100% | 0 | 96.64 | [WVP82966.1](https://www.ncbi.nlm.nih.gov/protein/WVP82966.1?report=genbank&log$=prottop&blast_rank=34&RID=9YRNT7GX016) |
| virion structural protein [Enterococcus phage vB_Efs10_KEN05] | 644 | 644 | 100% | 0 | 91.72 | [WZP35042.1](https://www.ncbi.nlm.nih.gov/protein/WZP35042.1?report=genbank&log$=prottop&blast_rank=35&RID=9YRNT7GX016) |
| tail tip protein Tal [Enterococcus phage SAP6] | 644 | 644 | 100% | 0 | 91.72 | [YP_009604019.1](https://www.ncbi.nlm.nih.gov/protein/YP_009604019.1?report=genbank&log$=prottop&blast_rank=36&RID=9YRNT7GX016) |
| tail fiber protein [Enterococcus phage 47] | 643 | 643 | 100% | 0 | 91.72 | [UOX39220.1](https://www.ncbi.nlm.nih.gov/protein/UOX39220.1?report=genbank&log$=prottop&blast_rank=37&RID=9YRNT7GX016) |
| tail tip protein Tal [Enterococcus phage BC611] | 643 | 643 | 100% | 0 | 91.41 | [YP_006488752.1](https://www.ncbi.nlm.nih.gov/protein/YP_006488752.1?report=genbank&log$=prottop&blast_rank=38&RID=9YRNT7GX016) |
| hypothetical protein [Enterococcus phage vB_EfaS_Ef7.1] | 643 | 643 | 100% | 0 | 91.72 | [QBZ69423.1](https://www.ncbi.nlm.nih.gov/protein/QBZ69423.1?report=genbank&log$=prottop&blast_rank=39&RID=9YRNT7GX016) |
| hypothetical protein [Enterococcus phage vB_EfaS_Ef2.2] | 641 | 641 | 100% | 0 | 91.1 | [QBZ69233.1](https://www.ncbi.nlm.nih.gov/protein/QBZ69233.1?report=genbank&log$=prottop&blast_rank=40&RID=9YRNT7GX016) |
| minor structural protein [Enterococcus phage vB_EfaS_PHB08] | 638 | 638 | 100% | 0 | 91.1 | [QBX32949.1](https://www.ncbi.nlm.nih.gov/protein/QBX32949.1?report=genbank&log$=prottop&blast_rank=41&RID=9YRNT7GX016) |
| virion structural protein [Enterococcus phage EF_WCK] | 348 | 348 | 54% | 1.00E-122 | 92.66 | [WVP82836.1](https://www.ncbi.nlm.nih.gov/protein/WVP82836.1?report=genbank&log$=prottop&blast_rank=42&RID=9YRNT7GX016) |
| virion structural protein [Enterococcus phage EF_PFS] | 317 | 317 | 47% | 4.00E-111 | 97.44 | [WVP83247.1](https://www.ncbi.nlm.nih.gov/protein/WVP83247.1?report=genbank&log$=prottop&blast_rank=43&RID=9YRNT7GX016) |
| virion structural protein [Enterococcus phage EF_WCK] | 317 | 317 | 47% | 4.00E-111 | 97.44 | [WVP82837.1](https://www.ncbi.nlm.nih.gov/protein/WVP82837.1?report=genbank&log$=prottop&blast_rank=44&RID=9YRNT7GX016) |
| virion structural protein [Enterococcus phage EF_TR2] | 316 | 316 | 48% | 1.00E-110 | 95.57 | [WVP83508.1](https://www.ncbi.nlm.nih.gov/protein/WVP83508.1?report=genbank&log$=prottop&blast_rank=45&RID=9YRNT7GX016) |
| virion structural protein [Enterococcus phage EF_PFS] | 307 | 307 | 46% | 4.00E-107 | 95.36 | [WVP83246.1](https://www.ncbi.nlm.nih.gov/protein/WVP83246.1?report=genbank&log$=prottop&blast_rank=46&RID=9YRNT7GX016) |
| minor structural domain protein [Enterococcus phage EF-P10] | 314 | 314 | 46% | 3.00E-104 | 96.03 | [AQT27698.1](https://www.ncbi.nlm.nih.gov/protein/AQT27698.1?report=genbank&log$=prottop&blast_rank=47&RID=9YRNT7GX016) |
| virion structural protein [Enterococcus phage EF_TR2] | 313 | 313 | 46% | 9.00E-104 | 95.36 | [WVP83507.1](https://www.ncbi.nlm.nih.gov/protein/WVP83507.1?report=genbank&log$=prottop&blast_rank=48&RID=9YRNT7GX016) |
| minor structural protein [Enterococcus phage EF-P10] | 175 | 175 | 26% | 1.00E-56 | 95.35 | [AQT27700.1](https://www.ncbi.nlm.nih.gov/protein/AQT27700.1?report=genbank&log$=prottop&blast_rank=49&RID=9YRNT7GX016) |
| minor structural protein [Enterococcus phage EF-P10] | 117 | 117 | 17% | 1.00E-34 | 98.25 | [AQT27699.1](https://www.ncbi.nlm.nih.gov/protein/AQT27699.1?report=genbank&log$=prottop&blast_rank=50&RID=9YRNT7GX016) |
| minor structural domain protein [Enterococcus phage EF-P10] | 51.6 | 51.6 | 7% | 2.00E-10 | 96 | [AQT27701.1](https://www.ncbi.nlm.nih.gov/protein/AQT27701.1?report=genbank&log$=prottop&blast_rank=51&RID=9YRNT7GX016) |

**Table S4. Description of ViPTree proteomic analysis results of phage vB_EFaS_ZC1.**

| **ID** | **length** | **taxid** | **name** | **host_group** | **score** | **SG to PP271740** | **%**  **.mean.idt** | **%**  **.len** |
| --- | --- | --- | --- | --- | --- | --- | --- | --- |
| PP271740 | 58596 | - | Enterococcus phage vB_EFaS_ZC1 | - | 38581 | 1 | 100 | 100 |
| LC547238 | 56582 | - | Enterococcus phage phiM1EF2 | - | 32719.5 | 0.8794 | 92.6 | 92.7 |
| MW677132 | 56099 | - | Enterococcus phage EFC1 | - | 31955.5 | 0.8736 | 91.8 | 92.3 |
| OL505085 | 56564 | - | Enterococcus phage EFap05-1 | - | 32477 | 0.8736 | 93.1 | 92.2 |
| MN939408 | 58127 | - | Enterococcus phage vB_EfaS_TV16 | - | 33112.5 | 0.866 | 92.4 | 94.2 |
| ON113173 | 57735 | - | Enterococcus phage vB_OCPT_CCS2 | - | 32653 | 0.8639 | 93.1 | 93.1 |
| OP172798 | 52450 | - | Enterococcus phage EF653P1 | - | 29689 | 0.8602 | 93.1 | 84.2 |
| OQ297175 | 59246 | - | Enterococcus phage vB_EfKS5 | - | 33168 | 0.8597 | 93.5 | 93.4 |
| MN871443 | 58036 | 2698989 | Enterococcus phage EfsWh_1 | Bacillota | 33104 | 0.858 | 92.6 | 94.4 |
| ON113177 | 57485 | - | Enterococcus phage vB_OCPT_PG2 | - | 32342.5 | 0.8577 | 91.4 | 94.3 |
| ON113171 | 55880 | - | Enterococcus phage vB_OCPT_SDS1 | - | 31487.5 | 0.8574 | 91 | 91.7 |
| MH618488 | 57811 | 2283014 | Enterococcus phage vB_EfaS_HEf13 | Bacillota | 32976.5 | 0.8547 | 93.7 | 92.4 |
| ON113178 | 57478 | - | Enterococcus phage vB_OCPT_PG9 | - | 32189 | 0.8503 | 90.9 | 93.8 |
| OP172800 | 56467 | - | Enterococcus phage EF653P5 | - | 31323 | 0.8442 | 92.4 | 89.7 |
| OP172799 | 56519 | - | Enterococcus phage EF653P3 | - | 31323 | 0.8434 | 92.4 | 89.7 |
| NC_041959 | 57081 | 1351735 | Enterococcus phage IMEEF1 | Bacillota | 32229 | 0.8354 | 91.9 | 92.4 |
| ON113180 | 57775 | - | Enterococcus phage vB_OCPT_PG13 | - | 31515 | 0.8333 | 90.6 | 92.4 |
| KY303907 | 58984 | 1932891 | Enterococcus phage EF-P29 | Bacillota | 31884.5 | 0.8264 | 89.7 | 93 |
| NC_021868 | 58305 | 1208587 | Streptococcus phage SP-QS1 | Bacillota | 31816 | 0.8247 | 91.4 | 91.6 |
| PP092045 | 57851 | - | Enterococcus phage EF_TR1 | - | 31046.5 | 0.822 | 90.7 | 89.5 |
| PP063012 | 57846 | - | Enterococcus phage EF_KTM | - | 31001.5 | 0.8206 | 90.5 | 89.7 |
| PP063009 | 57851 | - | Enterococcus phage EF_CW | - | 30975 | 0.8195 | 90.6 | 89.5 |
| PP063011 | 57857 | - | Enterococcus phage EF_PFS | - | 31000 | 0.8192 | 90.7 | 89.5 |
| PP063008 | 57854 | - | Enterococcus phage EF_CM | - | 30801 | 0.8152 | 90.6 | 89.7 |
| NC_029016 | 58000 | 1747287 | Enterococcus phage vB_EfaS_IME198 | Bacillota | 31366 | 0.813 | 90.2 | 91.4 |
| PP063007 | 57853 | - | Enterococcus phage EF_WCK | - | 30648 | 0.8123 | 90.5 | 89.6 |
| PP063013 | 57851 | - | Enterococcus phage EF_TR2 | - | 30731 | 0.812 | 90.5 | 89.5 |
| NC_041960 | 58619 | 1073766 | Enterococcus phage SAP6 | Bacillota | 31195.5 | 0.8086 | 89.5 | 91.8 |
| PP063010 | 57860 | - | Enterococcus phage EF_FB | - | 30580.5 | 0.8082 | 90.5 | 89.1 |
| PP028463 | 57848 | - | Enterococcus phage EF_RCK | - | 30637.5 | 0.8074 | 90.5 | 89.5 |
| MZ333457 | 57270 | - | Enterococcus phage SSsP-1 | - | 30107.5 | 0.8032 | 88.6 | 89.1 |
| MK721194 | 58018 | 2546636 | Enterococcus phage vB_EfaS_Ef7.1 | Bacillota | 30937 | 0.8019 | 90.2 | 90.5 |
| OP831581.2 | 58343 | - | Enterococcus phage EFKL | - | 30601.5 | 0.801 | 89.7 | 90.1 |
| KY472224 | 57408 | 1958912 | Enterococcus phage EF-P10 | Bacillota | 30338 | 0.7863 | 89.5 | 88.4 |
| MK800154 | 58938 | 2583507 | Enterococcus phage Entf1 | Bacillota | 30110.5 | 0.7804 | 88.5 | 88.4 |
| ON113179 | 57092 | - | Enterococcus phage vB_OCPT_PG11 | - | 28551 | 0.7628 | 86.6 | 86.1 |
| NC_024212 | 55726 | 1458851 | Enterococcus phage VD13 | Bacillota | 29425.5 | 0.7627 | 88.8 | 87.2 |
| MK570225 | 55244 | 2562559 | Enterococcus phage vB_EfaS_PHB08 | Bacillota | 29323 | 0.76 | 88.1 | 87.9 |
| NC_041861 | 55073 | 1458851 | Enterococcus phage VD13 | Bacillota | 29255.5 | 0.7583 | 88.8 | 87.1 |
| OL870612 | 56144 | - | Enterococcus phage UTI-EfS7 | - | 27982 | 0.7575 | 85.3 | 86.2 |
| NC_018086 | 53996 | 1173135 | Enterococcus phage BC611 | Bacillota | 28598.5 | 0.7413 | 89.7 | 83.6 |
| ON113172 | 57457 | - | Enterococcus phage vB_OCPT_SDS2 | - | 27337 | 0.7304 | 85 | 83.8 |
| ON113174 | 57118 | - | Enterococcus phage vB_OCPT_CCS3 | - | 27251.5 | 0.7268 | 84.7 | 84.2 |
| ON113175 | 57304 | - | Enterococcus phage vB_OCPT_Toy | - | 27010 | 0.7229 | 82.9 | 85.4 |
| MN103542 | 55876 | 2601631 | Enterococcus phage vB_EfaS_EF1c55 | Bacillota | 26633.5 | 0.6903 | 82.9 | 84.3 |
| MK721189 | 58400 | 2546635 | Enterococcus phage vB_EfaS_Ef2.2 | Bacillota | 26081.5 | 0.676 | 81.2 | 84 |
| ON086985 | 57289 | - | Enterococcus phage 47 | - | 25187.5 | 0.6721 | 80.4 | 82.5 |

**References**

1. Faul, F., et al., *G*Power 3: a flexible statistical power analysis program for the social, behavioral, and biomedical sciences.* Behav Res Methods, 2007. **39**(2): p. 175-91.

2. Dutka-Malen, S., S. Evers, and P. Courvalin, *Detection of glycopeptide resistance genotypes and identification to the species level of clinically relevant enterococci by PCR.* Journal of clinical microbiology, 1995. **33**(1): p. 24-27.

3. Willems, R.J., et al., *Variant esp gene as a marker of a distinct genetic lineage of vancomycinresistant Enterococcus faecium spreading in hospitals.* The Lancet, 2001. **357**(9259): p. 853-855.

4. Su, Y., et al., *Nucleotide sequence of the gelatinase gene (gelE) from Enterococcus faecalis subsp. liquefaciens.* Infection and immunity, 1991. **59**(1): p. 415-420.

5. Galli, D., F. Lottspeich, and R. Wirth, *Sequence analysis of Enterococcus faecalis aggregation substance encoded by the sex pheromone plasmid pAD1.* Molecular microbiology, 1990. **4**(6): p. 895-904.

6. Coque, T.M., et al., *Incidence of hemolysin, gelatinase, and aggregation substance among enterococci isolated from patients with endocarditis and other infections and from feces of hospitalized and community-based persons.* Journal of Infectious Diseases, 1995. **171**(5): p. 1223-1229.

7. Rice, L.B., et al., *A potential virulence gene, hyl Efm, predominates in Enterococcus faecium of clinical origin.* The Journal of infectious diseases, 2003. **187**(3): p. 508-512.
